# Multigenic resistance to *Xylella fastidiosa* in wild grapes (*Vitis* sps.) and its implications within a changing climate

**DOI:** 10.1038/s42003-023-04938-4

**Published:** 2023-05-30

**Authors:** Abraham Morales-Cruz, Jonas Aguirre-Liguori, Mélanie Massonnet, Andrea Minio, Mirella Zaccheo, Noe Cochetel, Andrew Walker, Summaira Riaz, Yongfeng Zhou, Dario Cantu, Brandon S. Gaut

**Affiliations:** 1 U.S. Department of Energy, Joint Genome Institute, Lawrence Berkeley National Lab, Berkeley, CA 94720 USA; 2 Dept. of Ecology and Evolutionary Biology, University of California, Irvine, CA USA; 3 Dept. of Viticulture and Enology, University of California, Davis, CA USA; 4 San Joaquin Valley Agricultural Center, United States Dept of Agriculture, Parlier, CA USA; 5 Agricultural Genomics Institute at Shenzhen, Chinese Academy of Agricultural Sciences, Shenzhen, China; 6 Present Address: Agricultural Genomics Institute at Shenzhen, The Chinese Academy of Agricultural Sciences, No. 7 Pengfei Road, Shenzen, 518120 China; 7 Present Address: Dept. of Viticulture and Enology, One Shields Avenue, University of California Davis, Davis, CA 95616-5270 USA; 8 Present Address: Dept. of Ecology and Evolutionary Biology, 321 Steinhaus Hall UC Irvine, Irvine, CA 92617-2525 USA

**Keywords:** Population genetics, Quantitative trait, Climate-change ecology

## Abstract

*Xylella fastidiosa* is a bacterium that infects crops like grapevines, coffee, almonds, citrus and olives. There is little understanding of the genes that contribute to plant resistance, the genomic architecture of resistance, and the potential role of climate in shaping resistance, in part because major crops like grapevines (*Vitis vinifera*) are not resistant to the bacterium. Here we study a wild grapevine species, *V. arizonica*, that segregates for resistance. Using genome-wide association, we identify candidate resistance genes. Resistance-associated kmers are shared with a sister species of *V. arizonica* but not with more distant species, suggesting that resistance evolved more than once. Finally, resistance is climate dependent, because individuals from low ( < 10 °C) temperature locations in the wettest quarter were typically susceptible to infection, likely reflecting a lack of pathogen pressure in colder climates. In fact, climate is as effective a predictor of resistance phenotypes as some genetic markers. We extend our climate observations to additional crops, predicting that increased pathogen pressure is more likely for grapevines and almonds than some other susceptible crops.

## Abstract (web summary)

Potential resistance genes for Xylella fastidiosa in wild grapes are identified and resistance is shown to be climate dependent.

## Introduction

Climate change is impacting crop yields by shifting temperatures, weather extremes, and water availability^1^, thereby affecting the distribution of arable lands^2^. There is, however, another important effect of climate change, which is the altered distribution of plant pathogens^3,4^. One especially prominent pathogen is the bacterium *Xylella fastidiosa* (Wells). *X. fastidiosa* is a generalist that colonizes > 300 plant species^5,6^, but it is pathogenic on major crops like citrus, coffee, almonds and grapevines (*Vitis vinifera* L. ssp. *vinifera*). Until recently, *X. fastidiosa* had been limited to the Americas, but human-mediated migration has led to its colonization of Europe, where it causes > ~$100 M of damage per year to the olive (*Olea europaea* L.) industry^7^. This olive example illustrates that the bacterium is more than a persistent threat in the Americas; it is also an emerging and expanding global threat to Europe, the Middle East^8^ and beyond^9^. Accordingly, there are urgent needs to better understand the genetic mechanisms of plant resistance^10^, particularly in the wild where both pathogens and hosts evolve^11^.

Thus far, studies of *X. fastidiosa*-mediated diseases have focused primarily on citrus^12,13^ and on Pierce’s Disease (PD) in domesticated grapevines, but also with an increasing emphasis on olives^14,15^. In grapevines, PD manifests by colonizing the xylem, leading to vascular blockages and eventual plant death after several years. In the course of infection, PD causes other detrimental symptoms, including marginal leaf necrosis, berry desiccation, irregular maturation of canes and abnormal petiole abscission^16^. The bacterium is spread from plant to plant by xylem-feeding insect vectors, which affect the severity and spread disease. The distribution of these insect vectors is being affected by changing climate^17^ and by anthropomorphic activity. One pertinent example is the glassy-winged-sharpshooter (GWSS; *Homalodisca vitripennis* Germar), which was introduced to Southern California in the late 1990s. The GWSS has a higher transmission efficiency compared to native vectors and fueled a large PD outbreak that has permanently altered viticulture in the region.

Although all domesticated grapevines are susceptible to PD, some wild relatives of grapevines segregate for PD resistance, likely reflecting the evolution of resistance in regions of persistent *X. fastidiosa* pressure^18^. Among wild grapevines, *Vitis arizonica* (Engelm.) merits particular interest because it exhibits strong resistance to PD and because it contains the only characterized plant locus to segregate for *X. fastidiosa* resistance, the *Pierce’s disease resistance 1* (*PdR1*) locus^19,20^. *PdR1* was identified by genetic mapping of a segregating family, defined by simple-sequence-repeat (SSR) markers, and backcrossed into susceptible grapevine cultivars to introduce resistance^21^. A recent study utilized BAC sequences of the region to identify candidate genes for resistance^22^. Two canonical leucine-rich receptor (LRR) loci were transformed into *V. vinifera*, but neither conferred resistance^22^. Additional candidate resistance genes have been identified based on comparative transcriptomics and proteomics in *V. vinifera*^23,24^, olives^15^ and citrus (*Citrus reticulata* Blanco)^25^.

Despite the enormous economic impact of *X. fastidiosa* infection, the genomic architecture of resistance has not yet been investigated in any species, and the genomic basis of resistance remains unclear. Here we address this shortcoming by performing genome-wide association (GWA) analyses for *X. fastidiosa* resistance in *V. arizonica*. In addition to identifying several novel candidate genes for resistance in *PdR1* and in other genomic regions, our work begins to fill another surprising gap. Although GWA and similar approaches are commonly used to study disease resistance in crops, surprisingly few studies have focused on the wild relatives of crops^11^. [One notable exception is the wild relative of soybean, *Glycine soja* (Siebold & Zucc.)^26,27^.] This dearth of studies is surprising both because crop wild relatives are a proven and valuable source of resistance genes for crop improvement^28^ and because studying resistance in wild samples may provide insights into the evolution of resistance and the ecological and climatic factors that shape resistance^11^.

In this study, we generate landscape genomic data from a sample of *V. arizonica* from throughout its native range and perform GWA based on a resistance phenotype - i.e., bacterial load after experimental inoculation. In doing so, we identify several genomic regions, including the *PdR1* region, that are associated with resistance, and we identify candidate genes in these regions based on an improved *V. arizonica* reference genome. We combine GWA with several types of evidence – including population genetic analyses, gene expression assays, comparisons among wild *Vitis* species, data from *V. vinifera* cultivars bred for PD resistance and bioclimatic modeling - to address three sets of questions. First, which and how many genic regions contribute to resistance, and what are some of the likely candidate resistance genes within these regions? Second, are these regions implicated in resistance across *Vitis* species and also in cultivars that were specifically bred for PD resistance? What do these inter-species analyses imply about the origin of resistance? Finally, does plant resistance correlate with climate? If so, what might this correlation imply about the potential effects of climate change? Overall, our work provides information about the genetics, evolution and ecology of PD resistance, all of which will help inform strategies to manage an economically damaging and expanding pathogen^29^.

## Results

### Genome-wide associations for resistance to Pierce’s Disease

**Fig. 1:**
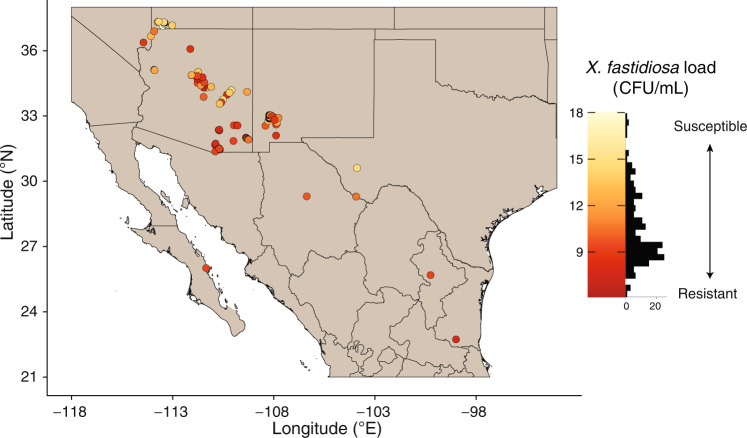
*Vitis arizonica* sampling and phenotypes. A map of the Southwestern United States and Northern Mexico indicates sampling locations of the *n* = 167 *V. arizonica* accessions used in this study. The color of sample locations (circles) are colored according to their resistance phenotype, as measured by bacterial load (CFU/mL). The histogram of phenotypes (in CFU/mL) is to the right of the map. Map generation relied on information from GADM, a publicly available database (http:gadm.org).

We studied the genetics of PD resistance in *V. arizonica* by combining three sources of information: an updated reference genome (accession b40-14, which is homozygous for PD resistance)^30^, whole-genome resequencing data from 167 accessions sampled across the species’ native range (Supplementary Fig. S1), and previously published PD resistance data measured in a common greenhouse environment on the same set of 167 accessions^30,31^. We used PD resistance as a quantitative variable - i.e., the log-transformed number of colony forming units (CFUs/mL) 12-14 weeks after experimental *X. fastidiosa* ssp. *fastidiosa* (Wells) inoculations (Supplementary Data S1). However, following precedence^31^, we also characterized individual accessions as resistant if they had *X. fastidiosa* concentrations below 13.0 CFUs/ml. Based on this threshold, our sample contained 135 resistant and 32 susceptible individuals, with the susceptible individuals more common in the northern region of the geographic distribution (Fig. 1).

**Fig. 2:**
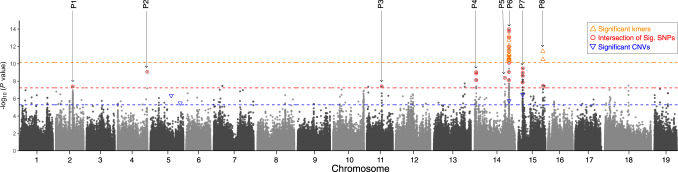
A Manhattan plot of the *V. arizonica* genome showing markers associated with bacterial load. The plot denotes each of the 19 chromosomes for haplotype 1. Each circle represents a SNP with a corresponding *p* value, based on EMMAX genome-wide association analysis. The 25 SNPs that were detected in two separate GWA analyses are circled in red and define the 8 peaks of association, which are numbered as P1, P2, etc., and referred to in the text. In addition to SNPs, the locations of significantly associated kmers and CNVs are provided when they overlap with a SNP-defined peak. The colored horizontal lines represent the cut-off p-values (*P* < 0.05, Bonferroni corrected) for the different marker types. Significant (*P* < 0.05, Bonferroni corrected) kmers and CNVs are represented by red and blue triangles, respectively.

We first performed genome-wide association (GWA) analyses based on SNP variants. To do so, we mapped resequencing data to the reference haplotype of the phased diploid genome and then tested for associations between high-quality SNPs and PD resistance using LFMM2^32^ and EMMAX^33^, both of which correct for genetic structure (see Methods). On the reference haplotype (hap 1), we identified 74 and 40 associated SNPs (Bonferroni *p* < 0.05) with the two methods, of which 25 were significant with both methods. We used these 25 SNPs to conservatively define eight peaks across five chromosomes (Fig. 2, Supplementary Figs. S2-S4, Supplementary Data S2). The most evident peaks were on chromosomes 14 and 15, with one of the former located between the SSR markers that define the *PdR1* locus. We also called SNPs independently to the second haplotype (hap2) and identified 11 significant SNPs in five peaks (Supplementary Figs. S5-S7, Supplementary Dataset 2). One of these peaks was also on chromosome 14 between the *PdR1* flanking markers.

Previous studies have suggested that *PdR1* alleles differ in size among *V. arizonica* accessions^34^, potentially implicating structural variants in resistance. We therefore investigated associations using copy number variants (CNVs), identifying 14,294 CNVs throughout the genome, of which 60 were in the 8 PD-significant peaks (Supplementary Data S3). The 60 CNVs included 19 deletions and 41 duplications, with means of 1.3 and 3.3 copies. We also performed GWA on the complete CNV set, finding four that were significantly (Bonferroni *p* < 0.05) associated with bacterial load (Fig. 2, Supplementary Figs. S8-S9). Two of these four CNVs mapped to two SNP-defined peaks: CNV10605 within *PdR1* (mean copy Number= 0.88, size= 6 kb, R = −0.36, *p* = 1.77e-06) and CNV10806 within peak 7 of chromosome 15 (mean copy number = 2.83, size = 17 kb, R = −0.38, p = 3.61e-07)(Fig. 2). The negative correlations for both CNVs indicated that a higher number of copies had lower bacterial loads and higher PD resistance. However, both CNVs had homology to long-terminal repeat transposable elements and so provided few insights into the functional basis of resistance. To further account for potential structural variation among accessions, we also applied GWA to 31 bp kmers, using a reference-free approach^35^ (Fig. 2, Supplementary Fig. S10). Of 115 significant kmers (Bonferroni *p* < 0.05) (Supplementary Data S4), 79 mapped to the reference genome (Supplementary Data S5) and 62 mapped uniquely to either hap1 or hap2. Among the uniquely mapped kmers, 57 of 62 were located on hap1 near *PdR1* and five were in peak 8 on chromosome 15 (Fig. 2). Altogether, CNV and kmer analyses corroborated four of the eight SNP-based peaks, added further evidence of the role of SVs in PD resistance, and confirmed *PdR1* as a candidate locus^19,20^.

We examined annotated genes under the eight hap1 peaks, using peak boundaries defined by 100 kb windows, since genome-wide LD decayed to background levels (*r*^2^ < 0.05) within this distance (Supplementary Fig. S11). The eight peaks included 124 genes, and several had annotations that implied a role in plant immunity (Supplementary Data S6). For example, peak 4 included a gene inferred to have calmodulin-binding function (g226310), which is involved in the regulation of plant disease response through changes in phytohormone biosynthesis^36,37^. This peak also had a gene annotated as a “syntaxin of plants 41” gene (g226360) that is homologous to genes that act in plant resistance against bacterial pathogens^38^. At *PdR1* (peak 6), we identified 7 leucine-rich repeat receptor-like protein (LRR-RLP) genes, one LRR receptor-like protein kinase (RLK) gene, and one lysin motif (LysM) RLK gene, all gene types that are commonly involved in pathogen detection and initiate the plant response^39^. Peak 7 contained two nucleotide-binding site leucine-rich repeat proteins (NBS-LRR; g243780, and g243820), genes that typically detect pathogens and initiate a host response, as well as a gene annotated as a Phloem protein 2-like (PP2) protein, which have antimicrobial properties in cucumber^40^. We also identified eight genes of interest on chromosome 15 (peak 8). Two of those genes have functional annotations related to phytohormone interactions (g252710, Ethylene-responsive transcription factor CRF4, and g252790, Abscisic Acid Insensitive-like 1 or ABIL 1), and thus could in theory play a role in plant immunity. Another four genes in peak 8 were annotated as acidic endochitinases, which can provide defense against fungal pathogens^41^. Finally, we studied the potential gene function associated with the 36 significant kmers that did not map to the reference genome by assembling reads containing the kmers and then aligning the assemblies to the NCBI Transcript Reference Sequences (“refseq_rna”). Of the assembled contigs, 80% had high similarity to three specific Receptor-like proteins (RLPs) (“ XM_010648495.2”, “XM_034852027.1” and “XM_019224733.1”). Overall, the set of candidate genes suggest that multiple diverse functions may contribute to PD resistance, but with likely involvement of classic disease resistance (R) genes.

**Fig. 3:**
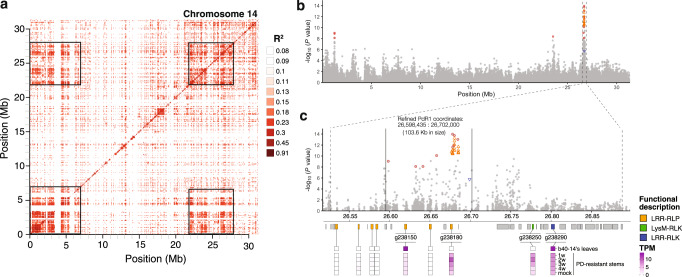
Genetic analyses of the *PdR1* region. **a** A plot of linkage disequilibrium (LD) across chromosome 14, where darker colors represent higher levels of LD. The two dark squares on the diagonal include GWA peak 4 (on the left, from 0 to 7 Mb on the chromosome) and the GWA peak that corresponds to PdR1 (on the right, from 22 to 28 Mb on the chromosome). The two off-diagonal squares reflect LD between these two distinct regions. **b** A Manhattan plot of chromosome 14 indicating the locations of peak 4 and the *PdR1* region. **c** An expanded representation of the *PdR1* region showing the location of significant SNPs (red circles denote SNP significant with two GWA methods), significant kmers (green triangles) and CNVs (blue triangles). The dashed vertical lines represent the 361 kb region defined by SSR markers and the 106 kb region defined by the location of significant markers. The schematics below represent genes in *PdR1* and a summary of gene expression results. Genes are denoted by rectangles and colored if they are related to R genes, with the category of R gene indicated by its color. Expression information shows expression, in transcripts per million (TPM) for leaves, for stems during four stages after infection and for mock controls.

We mapped previously used SSR markers to define the *PdR1* locus as a 361 kb region on hap1 chromosome 14 (with a corresponding 360 kb region on hap2), but we further characterized the locus in three ways. First, we evaluated linkage disequilibrium (LD) across chromosome 14. We observed two large blocks (~7 Mb in size) in high LD that contained the three PD-significant peaks of chromosome 14 (peaks 4, 5, and 6), even though peaks 5 and 6 were located on opposite ends of the chromosome from peak 4 (Fig. 3a). This striking pattern may simply reflect properties of our sample, but it also suggests that PD-related alleles co-segregate across peaks, implying that additive or epistatic interactions contribute to resistance in nature. Second, we focused on the location of significantly associated *PdR1* SNPs, which fell into a narrower 103.6 kb region containing six genes, three of which were RLPs (Fig. 3b, c).

Finally, we assayed gene expression in the region and manually confirmed gene annotations in this region with RNAseq data. One RLP gene (g238150) was expressed in b40-14 leaves, as was a receptor-like-kinase (RLK; g238290) that fell outside the 103.6 kb region (Fig. 3c). We also assayed gene expression in three resistant full-sibs that were inoculated with *X. fastidiosa* and a control (water). The stems above the inoculation site were sampled weekly for up to four weeks. Both g238150 and g238290 were expressed at higher levels than the control in at least one weekly stage, although not significantly so (*p* = 0.98 and *p* = 0.54 for g238150 and g238290). Two additional genes - an RLP (g238180) and an RLK (g239250) - also exhibited this pattern, and g238180 co-located with several associated kmers (Fig. 2). Altogether four candidate R genes (g238150, g238180, g238250, and g238290) under the *PdR1* peak were expressed in PD-resistant stems, but only g238150 and g238290 were found expressed in b40-14 leaves. All four of these genes were also present on the hap2 version of *PdR1*. Importantly, none of these four candidates were the closest homologs of the candidate genes that failed to confer resistance when transformed into *V. vinifera*^22^ (see Discussion).

### The genetic basis of resistance in breeding

The complex LD pattern on chromosome 14 suggests that resistance may require genic action from more than one locus - i.e., multigenic (horizontal) resistance. To investigate this possibility, we examined the distribution of kmers across accessions (Supplementary Data S7). Among the 117 kmers associated with bacterial load, 99 were common among resistant accessions; they were found in 65.0% of resistant plants, on average, but in only 9.5% of susceptible accessions. We labeled these kmers as resistant (R-kmers). In contrast, 16 kmers were detected in 67.2% and 10.1% of susceptible and resistant accessions, on average, suggesting associations with disease susceptibility (S-kmers). Interestingly, 10 of the 16 S-kmers mapped to a region on chromosome 15 that was ~12 kb upstream of a Jasmonic Acid-Amido Synthetase gene (*JAR1*, g252600). In Arabidopsis, changes in the expression of *JAR1* are associated with a reduction of host defenses^42^. We hypothesize that S-kmers are linked to variants that affect the expression of *JAR1* and promote susceptibility to *X. fastidiosa*. An important goal for breeding may be to avoid these S-kmers^43^.

**Fig. 4:**
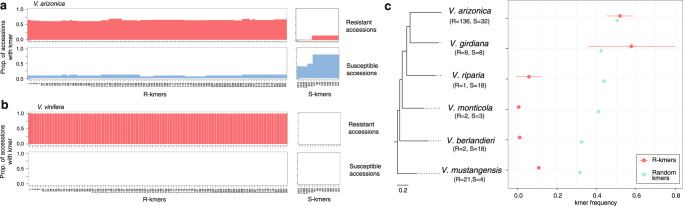
The presence of resistance and susceptibility kmers in different data sets. **a** Analyses within the *V. arizonica* sample set. The top-left graph indicates the 99 different resistance (R-kmers) kmers across the x-axis, with their detection frequency across the resistant (CFU/mL < 13) accessions. The top-right graph plots the average detection frequency of susceptibility kmers (S-kmers). The bottom-left and bottom-right graph are similar, they but show R-kmer and S-kmer detection frequencies among susceptible accessions. **b** The same graphs as in A, but the top graphs plot R-kmer and S-kmer detection frequencies for the five *V. vinifera* cultivars bred for PD resistance by backcrossing to *V arizonica*, while the bottom graphs represent susceptible *V. vinifera* cutlivars. **c**. Plots of kmer frequencies in six *Vitis* species. The species phylogeny is shown on the left, with the average detection frequency of R-kmers shown in red dot. The gray dots represent average detection frequencies of randomly chosen kmers that had similar population frequencies in *V. arizonica* as the set of R kmers. Whiskers denote 95% confidence intervals.

We then investigated the genomic content of five resistant cultivars (Ambulo Blanc, Caminante Blanc, Camminare noir, Errante noir and Paseante noir) derived from backcrosses to *V. arizonica* (accession b43-17)^44^ to test whether the basis of their resistance lay solely in *PdR1* or included additional genomic regions. After resequencing the five cultivars, we detected all 99 R-kmers in each cultivar but no S-kmers (Fig. 4b, Supplementary Data S8). In contrast, a control dataset from four susceptible *V. vinifera* cultivars (Cabernet Sauvignon cl. 08, Chardonnay cl. 04, Zinfandel cl. 03 and Petite Sirah) contained neither R-kmers nor S-kmers (Supplementary Data S8). Although our analyses used a reference (b40-14) that was not the source of PD resistance in backcrossed cultivars (b43-17), we found 56 kmers mapped to b40-14 hap1, 44 to hap2, and 53 to unplaced contigs. Importantly, the hap1 kmers mapped to both *PdR1* (51 kmers) and to peak 8 on chromosome 15 (5 kmers), suggesting these two regions contribute to resistance. As a complementary method, we scanned SNP heterozygosity in resistant cultivars, reasoning that backcrossed regions should be heterozygous for *V. arizonica*-specific alleles. As expected, this analysis revealed that portions of chromosome 14 were heterozygous across a region that encompassed Peak 5, Peak 6 (*PdR1*) and beyond (Supplementary Fig. S12). However, the proximal peak (peak 4) on chromosome 14 was also heterozygous (spanning from ~6.43Mbp to 6.59 Mbp on chromosome 14; Supplementary Fig. S12). Another prominent peak of heterozygosity on chromosome 9 did not correspond to peaks detected in our GWA. In short, both kmer and SNP analyses suggest that resistance backcrossed from *V. arizonica* encompassed multiple genomic regions.

### Resistance markers across *Vitis* species

These observations raise additional questions about the evolution of PD resistance: Did PD resistance arise only once in wild *Vitis* and, if so, is there evidence for the involvement of multiple genic regions? These questions are especially pertinent because all North American wild *Vitis* species can hybridize, with their genomes containing relics of introgression events that are enriched for RLK and RLP genes^30^. To address these questions, we extended kmer analyses to a population genomic sample of 105 individuals from six wild North American *Vitis*, all of which were assayed for resistance^30,31^ (Fig. 4c, Supplementary Data S8 & Supplementary Fig. S13). The six species were estimated to have a common ancestor ~25 million years ago (mya)^30^.

We hypothesized that PD resistance was introgressed across species and therefore predicted that the same R-kmers were present across species. We found (as expected) that R-kmers were at significantly higher frequencies in a subset of resistant vs. susceptible individuals for *V. arizonica* (Welch Two Sample WTS t-test, *p* = 4.50e-16), and also for its sister species, *V. girdiana* Munson (*p* = 0.007) (Supplementary Fig. S14). Interestingly, five of the R-kmers within *V. girdiana* mapped to the chromosome 15 peak, again suggesting a non-PdR1 component to resistance. These five kmers were detected in ~67% (12/18) of the *V. girdiana* individuals. These data suggest that *V. arizonica* and *V. girdiana* share the basis for resistance, either due to introgression or (more parsimoniously) common ancestry. For the remaining four species, no resistant individuals had > 50% of R-kmers (Fig. 4c), with no difference in R-kmer frequency between resistant and susceptible accessions (Supplementary Fig. S14). In fact, we detected R-kmers less often in these species than for a set of random *V. arizonica* kmers chosen to have similar population genetic frequencies as the R-kmers. Contrary to our hypothesis, the R-kmer distribution in these more distant species provide no evidence that the genetic mechanism of PD resistance (or at least the kmers linked to resistance) was introgressed from *V. arizonica/V. girdiana* to the remaining four species.

### Predicting PD resistance

**Fig. 5:**
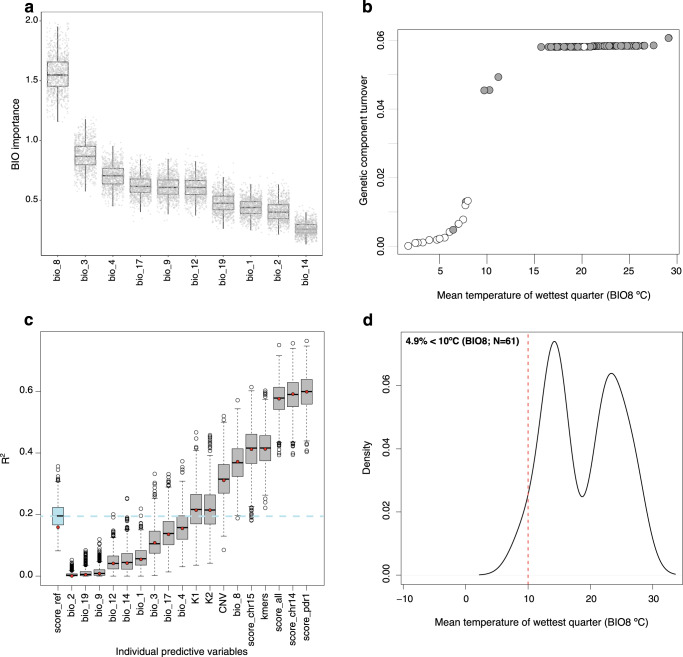
Relationships among resistance, genetic markers and bioclimatic data. **a** The estimated relative importance, from GF modeling, of each of the bioclimatic variables tested. The y-axis is a measure of the importance of various variables to explain the model - i.e., the relative importance of each bioclimatic variable for predicting changes in allele frequency across the landscape. Each boxplot denotes the average inferred importance of the bioclimatic variable, with the whiskers plotting the standard deviation of 1000 separate analyses (gray dots). BIO8 was estimated to have the biggest impact on the model in all 1000 analyses. **b** The turnover function showing the temperature range of BIO8 on the x-axis and the change in the genetic composition on the *y*-axis. The circles represent individuals that are colored by resistance (gray) or susceptible (white). **c** Individual predictors in a linear model to predict resistance levels (CFU/ml). The label score_ref represents sets of 1000 randomly chosen sets of 25 SNPs; K1 and K2 are the proportion of the assignments to each admixture group for each individual. The other predictors include bioclimatic variables and genomic data, as listed in the text, each evaluated 1000 times with bootstrapped datasets. Each boxplot reports the second and third quartiles, with median values in the square and circles showing outliers. The barplot whiskers report standard deviation, and the dashed horizontal line reflects the median value of 1000 replicates of the *R*_*pd*_ score. **d** The density distribution of BIO8 for a global database of locations of *Xylella fastidiosa* detection.

Because our plant accessions were sampled across a geographic range (Fig. 1), we can use the resequencing data to investigate relationships to climate. We utilized gradient forest (GF) to detect bioclimatic factors related to resistance. GF is a machine learning method that models the turnover in genetic composition and frequency across the climate landscape^45^ while identifying bioclimatic variables that are important to the construction of the model. As is common for GF applications^46^, we applied it to candidate SNPs, specifically the 25 SNPs associated with resistance. To test for robustness, we also repeated GF analysis 1000 times. In all 1000 runs, GF identified BIO8 (Mean Temperature of Wettest Quarter) as the most important model contributor among 10 bioclimatic variables, followed by BIO3 (Isothermality), BIO4 (Temperature Seasonality) and BIO17 (Precipitation of Driest Quarter) (Fig. 5a). Moreover, the turnover function revealed a bias in which susceptible individuals were from locations where BIO8 was <10 °C (Fig. 5b), which was confirmed by a significant pairwise comparison between resistant and susceptible individuals (Supplementary Fig. S15).

We performed two additional analyses to assess the generality between resistance and BIO8. First, we examined our complete dataset of all assayed *Vitis* individuals (*n* = 275) across six species. The dataset was highly skewed, because 30% of susceptible individuals – but only 1.6% of resistant individuals – had BIO8 < 10 °C (FET, *p* = 8.2e-12). This result held separately for *V. arizonica* (FET, *p* = 8.2e-12) and when *V. arizonica* was not included in the analysis (FET, p = 0.03). Second, we constructed a global dataset of known *X. fastidiosa* geographic locations that integrates across plant families and all *X. fastidiosa* subspecies^47^. Unfortunately, the dataset had few exact locations suitable for analysis, leaving only 61 reputable observations. Of these, fewer than 5% had BIO8 values < 10 °C (Supplementary Fig. S15), reflecting the previously reported relationship between temperature and *X. fastidiosa* presence^6,48–50^.

Given an association between plant resistance and temperature, we explored whether genetic or climatic factors better predicted bacterial load in *V. arizonica*. We assessed individual predictors with linear models, focusing on 10 bioclimatic predictors and nine genomic predictors (Fig. 5c). The genomic predictors included kmers, CNVs, assignments into genetic groups (K1 and K2), randomly chosen SNPs, and SNPs associated with PD. We summarized SNPs associated with PD with *S*_*pd*_, a measure that ranged from 0.0 to 1.0 and reflected the average proportion of alleles associated with resistance (where 0.0 is no resistance-associated alleles). Focusing on the performance of each of the 19 predictors, *S*_*pd*_ calculated across *PdR1* SNPs (10 total SNPs) had the strongest predictive power (R^2^ = 0.599), followed by related *S*_*pd*_ scores based on all candidate SNPs on chromosome 14 (16 SNPs, R^2^ = 0.592), all candidate SNPs across the genome (25 SNPs, R^2^ = 0.576) and finally all candidate SNPs on chromosome 15 (6 SNPs, R^2^ = 0.412) (Fig. 5c). Among the bioclimatic variables, BIO8 had an R^2^ of 0.370 in the linear model, which was much higher than the median value for 1000 randomly chosen sets of SNPs (R^2^ = 0.196) and similar to the predictive power of Kmers (R^2^ = 0.410) and CNVs (R^2^ = 0.307). Thus, BIO8 was a reasonable predictor of resistance, even in the absence of genetic data. Notably, the other bioclimatic variables that were identified by GF were not strongly predictive by themselves, e.g., BIO4, BIO17 and BIO3 had lower predictive power than random sets of SNPs (Fig. 5c).

## Discussion

*X. fastidiosa* causes Pierce’s Disease in domesticated grapevines (*V. vinifera*) and economically devastating diseases in other crops like citrus, olives, coffee and almonds^16^. A diverse body of work has investigated the basis of resistance across diverse crop species but has produced few plausible candidate resistance genes^15,22,23,25^. To date, however, no studies of *X. fastidiosa* resistance have taken advantage of full-scale genomic approaches like GWA. Indeed, GWA studies in the wild relatives of crops are surprisingly rare, despite the importance of understanding the basis of resistance in ecological settings^11^ and the transformative potential of such knowledge for crop breeding^28^. Here we have applied GWA to resistance in *V. arizonica*, based on an improved reference genome, on resequencing data from 167 wild-sampled accessions and on phenotypic data measured from *X. fastidiosa* infection assays performed in the greenhouse^30,31^. Together, these analyses have yielded information about genomic regions associated with PD resistance and also identified candidate genes within those regions. We have also studied the population frequencies of SNPs associated with resistance, using a machine-learning framework to help identify the climatic features that correlate with resistance and representing a novel extension of this approach to study interactions between resistance and climate.

Not surprisingly, GWA identified several significant SNP and kmer markers on chromosome 14 between the genetic boundaries that define the *PdR1* locus (Fig. 2). This locus was originally identified by genetic mapping and QTL analyses^19,51^ and it was subsequently backcrossed into susceptible *V. vinifera* to produce resistant cultivars^21^. There had been no insights into causative genes that lie within this region until Aguero et al.^22^ transformed two R genes separately into *V. vinifera*. They failed to find evidence for enhanced resistance using over-expression assays, but our study of a different accession (b40-14) provides further insights. First, based on careful genomic annotation combined with association and gene expression analyses, we have identified a narrower *PdR1* region with two strong candidates (g238150 and g238180), along with two additional candidates (g238250 and g238290) within the traditional *PdR1* locus (Fig. 3). Second, we have mapped the two candidate genes tested in Aguero et al. (RGA14 and RGA18) from b43-17^22^ to our genome. The closest homologues in the *PdR1* region were genes g238170, which encodes an putative LRR protein with 99.15% amino acid identity to RGA14, and g238120, which encodes a putative LRR-RLP with 92.46% identity to RGA18. Both g238120 and g238170 are located within the *PdR1* locus, but they are either not expressed or lowly expressed in leaves and stems across our sample of three PD-resistant full-sibs (Supplementary Data S9). Our results thus suggest that these genes, like their homologues in b43-17, are unlikely to be involved in PD resistance.

Overall, our candidate genes, which are present on both b40-14 haplotypes, differ substantially from those identified in b43-17. It is possible, of course, that different genes confer resistance in different accessions, given that structural variants are common in *Vitis* genomes^52^ and that allelic heterogeneity for resistance is also common^53^. A reasonable way to test our candidates would be to generate knock-outs in *V. arizonica*, but unfortunately transformation in *Vitis* is currently efficient only for a narrow set of *V. vinifera* cultivars^54^. Hence, an important challenge for viticulture is to improve transformation techniques for application to more cultivars and to agronomically valuable wild species. A future goal is to transform *V. vinifera* with sets of our candidate genes both as single loci and in combination, because disease resistance genes often act in tandem and also because stacking resistant genes is often desirable.

It is possible that candidate genes from *PdR1* will not be sufficient to confer complete resistance because contributions from additional genomic regions are required. There is evidence consistent with and against this multigenic hypothesis. For example, in an early study of PD resistance among *Vitis* species, Mortensen (1968) performed controlled hybrid crosses and concluded that complementary gene action among three independent genes best explained his results^55^. Similarly, although multiple QTL studies have identified *PdR1* as a major effect locus^19,20,56,57^, they also hint that other regions may be important. For example, a recent study used interval mapping and found that *PdR1* was the only major QTL, explaining 55.5% of phenotypic variation^57^, but phenotypic associations were also detected to regions of chromosomes 3, 7, 9, 10 and 15. Another study investigated the basis of resistance across 17 *V. arizonica* accessions and identified three accessions for which the *PdR1* region explained < 10% of phenotypic variation for resistance, suggesting that the possibility that resistance arises from non-*PdR1* locations in some accessions^56^.

The value of GWA is that, unlike QTL studies, it integrates across an extensive population sample. Here we have done so in the context of a platinum-level reference genome, which likely provides a more complete view of the basis for resistance. Our results are tentatively consistent with the idea that multiple genomic regions contribute to resistance. First, SNPs associated with resistance are found across the genome. We studied these significant SNPs further by measuring effect sizes - i.e., by estimating the slope of the phenotypic response to genotypic categories (Supplementary Fig. S16). We caution that this approach is subject to numerous caveats, including the Beavis effect and a lack of independence among SNPs, and thus must be viewed cautiously; ultimately effect estimation requires a second independent dataset^58^. Nonetheless, we find that the SNPs within peak 4 produce ~1.2-fold higher slopes, on average, than those within the *PdR1* peak (peak 5), which have similar (1.02x) slopes, to the SNPs in peak 6 on chromosome 15. Moreover, the SNPs with the largest inferred effect are not in *PdR1*, although *PdR1* has the most associated SNPs. Thus, this simple measure of effect sizes provides little evidence that SNPs in *PdR1* explain resistance phenotypes more completely than significant SNPs from other peaks and regions. To further explore this multigenic theme, we have also applied a multi-locus mixed model, which can more accurately control for false positives than some other methods (see Methods). Although the associations to *PdR1* remained, the highest proportion of significant SNPs were in other regions of the genomes, including significant hits on chromosomes 2, 4, and 11 (Supplementary Data S10 and Supplementary Fig. S17). We note, however, that some of our peaks are not recapitulated with this method.

Second, the striking LD pattern on chromosome 14 suggests the possibility of additive or epistatic interactions between distinct regions on the chromosome (Fig. 3a). Another explanation for this pattern could be reduced recombination on chromosome 14. However, there is no evidence for reduced recombination from genetic maps and QTL mapping in F1 populations^19,20^, which discriminate the *PdR1* region from the proximal end of the chromosome where peak 4 is located. The argument that this pattern arises from recombination is further discounted by the fact that the rest of the chromosome, including sequences intervening peaks 4 and 5, are not in strong LD (Fig. 3a). Ultimately, we cannot discriminate whether this unique pattern is caused by sampling phenomenon or even whether this pattern of LD contributes to our detection of peak 4. We speculate, however, that the pattern of LD is likely best explained by selection for resistance that acts on alleles in both regions. Clearly this speculation requires further investigation.

A third observation is based on the distribution of R-kmers across species. We found, for example, that *V. girdiana* and *V. arizonica* share R-kmers that map to at least two different chromosomes (14 and 15), suggesting that not only that multiple genomic regions contribute to resistance but that these regions may have contributed to resistance since the divergence of these two species ~ 23 million years ago^30^ (Fig. 4b). Finally, we have also investigated the genomics of five resistant *V. vinifera* cultivars, representing the only grapevine cultivars bred for PD resistance to date. Because these cultivars were produced by backcrossing, we hypothesized that genomic regions contributing to resistance are highly heterozygous. The *PdR1* region was highly heterozygous, as expected, but so were additional peaks on chromosomes 8, 9, 14 and 15 (Fig. 4a). These patterns suggest that additional, non-*PdR1* regions may have been instrumental for backcrossing resistance into the *V. vinifera* background and continue to hint that non-*PdR1* regions contribute to the resistance phenotype.

Inferring the spatial distribution of disease resistance is critical for understanding its evolution and ecology^53^. We have investigated resistance across the landscape of *V. arizonica* (Fig. 1) and across six wild species that segregate for PD resistance. Given that all North American *Vitis* species are interfertile and that there is ample genomic evidence for historical introgression of resistance genes among species^30^, we predicted that the genetic solution to PD would yield clear signals of introgression. As mentioned above, we find that R-kmers are commonly shared between *V. arizonica* and its closest species in our sample, *V. girdiana*, but not across the other species (Fig. 4c**)**. It is possible that we cannot detect introgression events because causative loci and associated R-kmers have become uncoupled over evolutionary time. At a minimum, however, our results provide no evidence that PD resistance has introgressed from *V. arizonica* to the non-*girdiana* species in our sample, as speculated previously^22^. If true, this implies that other wild *Vitis* species have independently evolved mechanisms of PD resistance, such that further study of these species may provide additional insights into alternative genetic mechanisms of PD resistance. In this vein, one particularly interesting species is *V. mustangensis* (syn *V. candicans*), which has not been widely utilized agronomically as a rootstock or for hybrid scion breeding, but it does segregate for PD resistance and also contains alleles that may be useful for viticulture in the context of climate change^59^.

We have taken advantage of our geographic sampling to investigate correlates between resistance and climate, finding that resistance-associated SNP markers correlate with climatic variables, especially temperature in the wettest quarter (BIO8). More specifically, susceptible plants tend to be found below specific BIO8 thresholds. Based on the turnover function, which is inferred from both climatic and population genetic data, the BIO8 threshold lies between 8 °C and 10 °C (Fig. 5b). Focusing specifically on the higher of these two thresholds, we have found that the 10 °C threshold not only applies within *V. arizonica*, but also to our expanded sample of *Vitis* species and to a wider (although still quite limited) geographic sample of *X. fastidiosa* that summarizes across plant hosts and bacterial subspecies^47^ (Fig. 5d). Somewhat remarkably, this simple climatic measure predicts bacterial load nearly as well as some genetic markers (e.g., kmers) and better than others (e.g., CNVs) (Fig. 5c). This is, to our knowledge, the first time that genomic data have been used to associate plant resistance with climate, yielding a useful bioclimatic predictor. Our findings are not without precedent, however, because temperature has previously been identified as a strong predictor of *X. fastidiosa* distribution and presence^6,48,49,60^. Combining these observations, we suspect that individuals with low (<10 °C) BIO8 temperatures lack resistance because *X. fastidiosa* growth is hampered at low temperatures^50,61^ and/or because temperature affects its insect vectors^17,62^. Plant resistance will not be favored by natural selection in regions where the pathogen does not persist, particularly if there is a fitness cost to resistance, as has been demonstrated for R-gene mediated resistance in *A. thaliana*^63^.

**Fig. 6:**
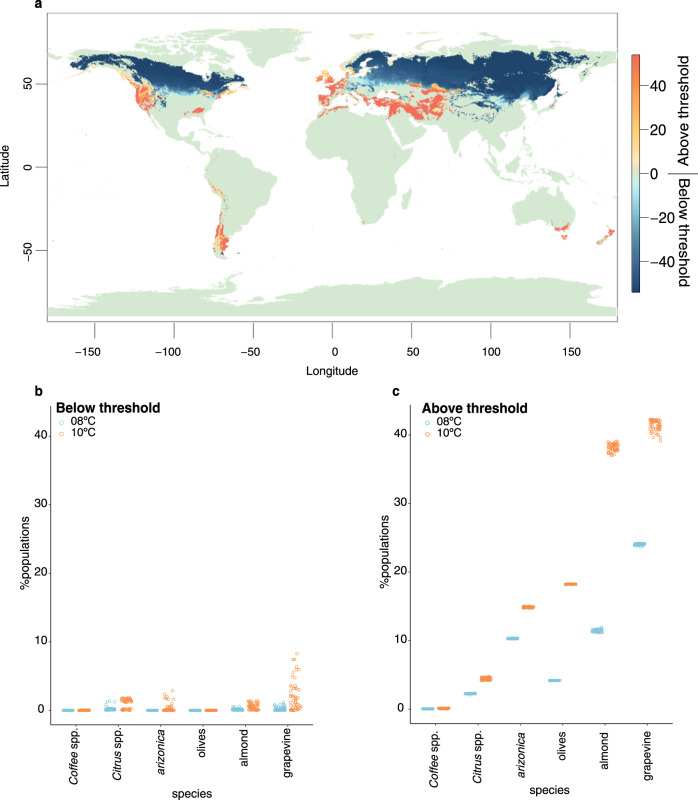
Climate predictions and projections of the prevalence of *X. fastidiosa* for focal crops. **a** The map portrays the number of climate models (out of 54 total) that support movement across the BIO8 = 10 °C threshold. The warmer colors reflect regions that are moving from below (in the present) to above the threshold, while the cooler colors portray areas that are moving from above (in the present) to below the threshold. The intensity of color in the scale bar reflects the number of 54 climate models that agree with the threshold transition. The generation of this map, as well as the maps in Figures S1 and S18, relied on publicly available information from WorldClim2 (https://www.worldclim.org/) and CMIP6 (https://pcmdi.llnl.gov/CMIP6/). **b** A summary of the percentage of locations associated with movement from above the 8 C °C or 10 °C threshold (in the present) to below that threshold for five crops and for *V. arizonica*. **c**. A summary of the percentage of locations associated with movement from above the threshold (in the present) to below the threshold for five crops and *V. arizonica*. Both b and c are based on 6204 locations for coffee; 3386 locations for almonds, 1111 locations for *V. arizonica*; 5256 locations for *Citrus*; 174,713 locations for olives and 33,225 locations for grapevines. In both b and c, each dot represents an estimate based on one of the 54 climate models.

Previous work has modeled the distribution of *X. fastidiosa* under climate change^6,48,49,60,64^, but these models have not been informed by data on the distribution of plant resistance. To illustrate how such information may be useful, we have employed climate models to predict where BIO8 will shift in the future. More specifically, we have identified regions where BIO8 will transition across the thresholds of 10 °C (Fig. 6a), as informed by our analysis of genetic variants associated with plant resistance. By categorizing regions where BIO8 is predicted to move from below (or above) 10 °C in the present to above (or below) 10 °C in the future, we can identify regions where *X. fastidiosa* pressure is likely to expand or contract. We performed these categorizations across 54 climate models to consider uncertainty in global circulation models, shifts in greenhouse gas emissions and time (see Methods). Summarizing across models, we have found that most of the globe will not transition - i.e., it will remain either above or below the 10 °C (Fig. 6a) during the wettest quarters. Perhaps unsurprisingly^65^, large portions of Canada, Eastern Europe, Russia and Northern Asia climate are predicted to move beneath the10 °C BIO8 threshold, suggesting that these regions may be less likely to have pathogen pressure in the future.

There are, however, discrete areas of the Western Americas, Western Europe, Central Asia, Southern Australia and elsewhere that are predicted to transition above the 10 °C (Fig. 6a) threshold in most models, suggesting increasing *X. fastidiosa* pressure in these regions. These models of course make numerous assumptions. Some are common to all climate models (e.g., a reliance on accuracy of the climate predictions) and to previous *X. fastidiosa* species distribution models (which assume *X. fastidiosa* is not dispersal limited and often ignore the potential of *X. fastidiosa* to evolve to new temperature regimes^46,64^). Some are more specific to this work, specifically that the BIO8 threshold has application to plant species beyond *V. arizonica*, but this assumption is supported by our analysis of data that includes different plant hosts (Fig. 5c). Finally, we note that there is some uncertainty in the appropriate BIO8 threshold, because the turnover function has inflection points along the range of 8 °C to 10 °C (Fig. 5b). Hence, we repeated these analyses with an 8 °C threshold, finding similar results (Supplementary Fig. S18). Importantly, our climate modeling illustrates how data about plant resistance can help inform the potential distribution of disease under a shifting climate.

In fact, we can proceed one step further by assessing the potential effects of climate on specific plant taxa: wild *V. arizonica* and five affected crops *(*grapes, coffee, almonds, citrus and olives). To do so, we first downloaded data on global locations where each species is grown, and we then used climate projections to estimate the proportion of locations that will transition over either the 8 °C threshold or the 10 °C BIO8 threshold under climate models (see Methods). Using this approach, we predict that few locations for these crops will transition below the 8 °C and the 10 °C thresholds. In citrus and grapes, for example, the average estimate across the 54 climate models is that only between 0.21% and 0.97% (of 7853) locations and between 0.24% and 2.10% (of 40,075) locations will transition below 8 °C or 10 °C, respectively, under climate projections (Supplementary Fig. S18 & Fig. 6b). Perhaps not unexpectedly, a much higher proportion of locations are estimated to exceed the 8 °C and 10 °C threshold over time. For example, we estimate that between 11% and 38% of regions of almond cultivation will transition above the 8 °C and 10 °C threshold, respectively (Fig. 6c). The corresponding numbers for grapevine cultivation are 24% and 41%, suggesting that roughly a quarter of viticulture will be subjected to additional *X. fastidiosa* pressure even under the less expansive value based on an 8 °C threshold. Similarly, 4% and 18% of olive growing locations are expected to transition above the 8 °C and 10 °C threshold, respectively, corroborating previous modeling work that predicts further spread of the bacterium amongst olive growing regions even under current climates^7^. We note that, like some other climate studies, our calculations treat *X. fastidiosa* subspecies similarly, even though there are differences in their host specificity^6,66^ and distributions^7,67^. However, *X. fastidiosa* can move between crops, as has been shown, for example, between almonds and grapevines^68^; hence we believe that a multi-crop perspective is critical.

Overall, our study has considered the genomic, climate and evolutionary context of resistance to a pathogen that is an emerging global pest and that already causes devastating economic damage to several crops. By studying the complex genetic architecture of PD resistance in a wild grapevine, we have implicated several genomic regions in resistance and identified genes that are fitting candidates for genetic introduction into susceptible crops. Furthermore, by comparing features across breeding lines and wild *Vitis* species we have uncovered hints about the origins of this critical trait. Finally, our work has highlighted the potential of combining landscape-scale resequencing data and climate models to predict the shifting pressures of a damaging plant pathogen. These results underscore the urgent need to identify additional *X. fastidiosa* management and containment methods, potentially via enhanced information about resistance mechanisms and genes.

## Methods

### Plant material and PD disease evaluations

We studied 167 accessions of *V. arizonica* collected across the southwestern states of the US and northern Mexico, covering the distribution of the species (Fig. 1 and Supplementary Fig. S1). The sampling location was available for all 167 *V. arizonica* accessions, which are part of a living collection of Southwest *Vitis* accessions maintained at the University of California, Davis; all *Vitis* accessions used in this paper were from that collection. PD resistance in these 167 accessions were previously assayed in controlled greenhouse trials^30,31^, following previously published protocols^31,69^. Briefly, accessions were inoculated with *Xylella fastidiosa* subsp*. fastidiosa (*Wells) using a randomized complete block design that included at least two water-inoculated plants and susceptible *V. vinifera* cultivar Chardonnay plants as controls. Nineteen screens were carried out from 2011 to 2020, with a minimum of four biological replicates per accession. Disease severity was assessed 10 to 14 weeks after inoculation using categorical phenotypes for disease severity. Whole immunoglobulin (IgG) antiserum raised against *X. fastidiosa* in a rabbit were used to quantify the bacteria levels from stem tissue above the inoculation site from 12 to 14 weeks, using a double-antibody sandwich ELISA method as described previously^69^. In this assay, absorbance values were converted to colonizing forming units per milliliter (CFU/ml) concentrations using a standard calibration curve. The ELISA data were log-transformed and statistical analysis was performed using JMP Pro14 software (Copyright 2020, SAS Institute Inc.) to determine the variability of ELISA for the reference control plants across experiments. Following precedent^30,31^, we used the least squared means of CFUs/ml across biological replications as an indicator of disease resistance. Lower bacterial load reflects higher PD resistance.

### Genomic reference, resequencing data and SNP calling

Version 1 of the *V. arizonica* sequence (b40-14 v1) was published previously^30,70^, but this study relied on an updated version (b40-14 v2). Version 2 was a complete re-assembly based on the application of Haplosync^71^ combined with ~2000 rhAmpSeq Vitis markers^72^, independent of any single reference genome assembly. This new version of *V. arizonica* genome chromosome haplotypes were better phased, with fewer unplaced sequences (~65 Mb; 3035 gene loci). Correct phasing was assessed during the assembly quality control steps based on genome and locus-specific markers and gene content. The gene annotations were reported between versions 1 and 2, so that genes may have moved locations in the new assembly but no new annotations were performed. The genome contains 57,003 gene loci. This version 2 assembly represents the most contiguous genome assembly of any wild *Vitis* genome released to date and was used as the reference for all analyses. The genome is available for browsing at *grapegenomics.com*, and the long-read sequencing data are available from NCBI BioProject ID PRJNA593045.

Our *V. arizonica* resequencing dataset consisted of short-read, whole genome data from 167 *V. arizonica* individuals, for which a subset of *n* = 20 had been sequenced previously^30^. The data for this subset was available from NCBI BioProject PRJNA731597. For the remaining 147 individuals, genomic DNA was extracted from leaf samples with the Qiagen DNeasy plant kit and assessed for quality and degradation on agarose gels and a BioAnalyzer (Agilent). Sequencing libraries were constructed with an insert size of ~300 bp using Illumina library preparation kits; the libraries were assessed for quality and quantified using a Kapa library quantification kit; libraries were then sequenced using the Illumina HiSeq 2500 platform with 2 × 150 bp paired reads to a target coverage of 10x. The raw sequencing data for this study were deposited in the Short Read Archive at NCBI under BioProject ID: PRJNA842753.

We filtered and evaluated raw reads from 167 individuals using Trimmomatic-0.36^73^ and FastQC^74^. Reads were scanned in windows of four base pairs, cutting when the average quality per base dropped below 20. We removed leading and trailing bases of reads that had a quality below 3 and finally kept reads with sizes of 60 bp or higher after trimming. Filtered reads were then mapped to the reference genome (independently to Hap1 and Hap2) with the BWA-MEM algorithm^75^ implemented in bwa-0.78. Joint SNP calling was conducted using the GATK v.4.2.2.0 pipeline^76^ for Hap1 and Hap2 independently. We first used the integrated version of Picard tools^77^ to remove duplicated reads with the “MarkDuplicates” function, followed by the “AddOrReplaceReadGroups” function to label the reads for each individual. For the SNP prediction we used the HaplotypeCaller algorithm with a sample ploidy of 2 and a mapping base quality score threshold of 20 (Q > 20). We combined the VCF files of all individuals to make the final SNP calls using the “GenotypeGVCFs” function with default parameters. We then filtered raw SNPs with bcftools v1.9^78^ (https://samtools.github.io/bcftools/) and vcftools v0.1.17 (https://vcftools.github.io/)^79^. We kept SNP sites for downstream analysis if they were biallelic, had quality higher than 30, had a depth of coverage higher than five reads, had no more than three times the median coverage depth across accessions, and had no missing data among individuals. Additionally, the following expression was applied under the exclusion argument of the filter function in bcftools: “QD < 2.0 | FS > 60.0 | MQ < 40.0 | MQRankSum < −12.5 | ReadPosRankSum < −8.0 | SOR > 3.0”.

### Population Structure and genome-wide associations

We transformed the VCF file into BEAGLE format using vcftools 0.1.17. We used BEAGLE files as input to evaluate the genetic structure of the *V. arizonica* using the NGSAdmix software included in the ANGSD package version 0.931-21-g13af014^80^. We ran NGSadmix for 1 to 10 ancestral populations (*K*), repeating analyses 10 times for each *K* value and including variants with a minimum minor allele frequency > 0.05 (Supplementary Fig. S19). We then employed the Cluster Markov Packager Across K (ANGSD package version 0.931-21-g13af014 software^81^ to detect the *K* value with the highest likelihood of *K* = 2.

The NGSadmix results were used to help guide controlling for genetic structure in genome-wide association (GWA). We performed GWA to identify significant associations between SNP allelic frequencies and bacterial load (*Xylella fastidiosa* CFU’s), using two different methods that control for population structure using different approximations. First, we used LFMM2, which uses latent factors to control for genetic structure^82^. The number of latent factors were chosen based on a visual observation of the screenplot of the percentage of variance explained by the loadings of the genetic PCA of all individuals. The PCA was obtained using all loci with no missing data and with the prcomp function in R^83^. In total, we defined K = 5 latent factors (Supplementary Fig. S20). Next, we ran LFMM2 using a ridge penalty (function lfmm_ridge), and we controlled for genomic inflation factor (function lfmm_test, with calibrate= “gif”). We corroborated that the *p* values had a flat distribution, and we corroborated that the genetic structure was well controlled based on a qqplot (Supplementary Figs. S4 & S7).

In addition, we used the variance component algorithm Efficient Mixed-Model Association eXpedited (EMMAX) version beta-07Mar2010^84^. We converted the set of filtered SNPs with no missing data from VCF format to transposed ped format using PLINK version 1.90b6.16^85^. Using the transposed ped files as input, we calculated the Balding-Nichols (BN) kinship matrix using the “emmax-kin” script and default parameters. Finally, we ran the associations using the SNPs data (as transposed ped), the BN matrix, and the phenotype as Least Squares Means of CFU/ml of *X. fastidiosa* for each accession. Both methods (EMMAX and LFMM2) were adjusted for multiple comparisons using the Bonferroni correction of the function “p.adjust” from the stats package version 4.1.2 in R. We focused only on SNPs and candidate regions that were detected by both methods.

Finally, we also used the R package mrMLM v 5.0.1^86^ to perform a multi-locus genome-wide association. We used the FASTmrEMMA algorithm^87^ within the mrMLM package with the same set of SNPs, bacterial load measurements and the kinship matrix described above to run multi-locus associations.

### Kmer-based GWA

To perform GWA based on kmers, we followed a previously published pipeline^35^ (https://github.com/voichek/kmersGWAS). Briefly, we extracted all kmers and canonized (i.e. reverse complement is assumed to be the same kmer) kmers of 31 bp in size using KMC version 3^88^. We extracted the kmers directly from the paired and unpaired filtered reads for each of the 167 *V. arizonica* samples independently. We compared the kmers across samples and created a table of kmers that were found in at least 5 individuals (“-mac 5”) and in each canonized/non-canonized form in at least 20% of individuals from which it appeared in (“-p 0.2”). We used the script “emma_kinship_kmers” included in the pipeline with a MAF < 0.05 filter to create a kinship matrix based on the kmer table. Finally, we ran the kmer-GWAS with the script “kmers_gwas.py” and GEMMA version 0.98.5^89^ with the kinship matrix, the kmer table, and the phenotype data as Least Squares Means of CFU/ml of *X. fastidiosa* for each accession. The script provided a list of 9991 kmers that passed a parametric test as an initial filter. To filter more stringently, we used the number provided in the file “pheno.tested_kmers”, which was 967066440, to adjust the *p*-values with a Bonferroni correction using the program “p.adjust” from the stats package version 4.1.2 in R. To create a textual version of the presence/absence kmers of the significant Kmers we used the “filter_kmers” from the pipeline. Given 115 significant kmers, we mapped them to the *V. arizonica* genome using BLASTN^90^ and a word size of 8 bp. We filtered all the BLASTN results and kept alignments, allowing a maximum of 1 mismatch.

We further explored the sequences of the 36 kmers that did not map to our reference genome. We first searched and extracted the reads matching these 36 kmers across the 167 *V. arizonica* accessions. We then used SPADES v3.15.4^91^ to assemble the matching reads into contigs independently for each kmer. Finally, we aligned the resulting contigs to the Reference RNA Sequences database (Refseq_rna) using BLASTN (visited on 06/10/2022) and recorded the top hit gene.

### Copy Number variation analysis and associations

To identify Copy Number Variants (CNVs), we used the program CNVcaller version 2.0 (https://github.com/JiangYuLab/CNVcaller)^92^. CNVcaller uses normalized read-depth values across windows in the genome to identify CNVs and it is especially suited for large population data like our *V. arizonica* sample. First, we generated a duplicated window record file specifically from our genome reference *V. arizonica* b40-14 v2 using a window size of 2 kb and for each chromosome independently. We then analyzed the individual read depth in 2 kb non-overlapping windows using the “Individual.Process.sh” script and the alignment files of all 167 accessions in bam format with the PCR duplicated reads removed during the SNP calling pipeline (see section above). The script produces normalized values of read depth across the genome for each genotype. We then used the normalized read depth values of all genotypes as input to the script “CNV.Discovery.sh”, excluding windows with a lower frequency of gain/loss individuals of 0.1 (“-f 0.1”) and with Pearson’s correlation coefficient lower than 0.3 (“-r 0.3”). Finally, we used “Genotype.py” to classify genotypes across the population according to their CNV profiles.

To explore the associations between CNVs and PD-resistance, we used the CNVcaller estimation of diploid copy number for each CNV and tested for correlations with *X. fastidiosa* bacterial levels, while taking into account the genetic structure of the *V. arizonica* population. We used the function “pcor.test” from the R library “ppcor” v1.1^93^ to run a partial correlation for each of the CNVs, using genotype assignment (*Qi*) values from the genetic structure analysis (see section above) as the confounding variable. To account for multiple testing we imposed a Bonferroni correction and identified significant CNVs with adjusted *p* < 0.05.

### Defining PD-associated peaks

We performed a total of four association analyses: LFMM2 based on SNPs, EMMAX based on SNPs, kmer-based GWA, and CNV-based GWA. From these analyses we defined eight GWA peaks of interest in the genome (Fig. 2). To define these peaks, we required that a peak contains at least one SNP that was significant with both LFMM2 and EMMAX. However, most peaks had multiple pieces of evidence - i.e., either more than one SNPs, significant kmers and/or CNV variants. When applying this logic, we focused only on kmers that mapped uniquely to the genome and so excluded 17 kmers that mapped to multiple places in the genome with the same identity and alignment length.

### Analyses of PD-associated kmers in other *Vitis* species

We identified kmers associated with resistance in *V. arizonica* sample and then characterized their presence in three different resequencing datasets: *i*) a multiple species *Vitis* dataset, *ii*) a dataset generated from scion cultivars bred for PD resistance by backcrossing to the b43-17 accession of *V. arizonica*, and *iii*) a set of PD susceptible cultivars. The first dataset included 105 accessions from five species: *V. arizonica* (*n* = 22), *V. candicans* (*n* = 24), *V. berlandieri* (*n* = 22), *V. girdiana* (*n* = 18) and *V. riparia* (*n* = 19)^30^. All of these accessions had been assayed for PD resistance, and categorized as resistant if CFU/mls were <13.0 at the time of assay. In this dataset, 20 *V. arizonica* were resistant (*n* = 20), 21 *V. candicans* (*n* = 25), 3 *V. berlandieri* (*n* = 21), 9 *V. girdiana* (*n* = 17), 2 *V. monticola* (*n* = 5) and 2 *V. riparia* (*n* = 20). The resistance assay data and sampling locations of the accessions are available^30^. For the second dataset, we generated resequencing data for five PD resistant cultivars (Ambulo Blanc, Caminante Blanc, Camminare noir, Errante noir and Paseante noir). DNA extraction, library preparation and Illumina sequencing followed the protocols mentioned above, and the data were deposited into Bioproject: PRJNA842753. Finally, the ‘control’ dataset of PD susceptible accessions was downloaded from public databases (Cabernet Sauvignon cl. 08: SRR3346862; Chardonnay cl. 04: SRR5627799; Zinfandel cl. 03: SRR8727823; and Petite Sirah: SRR12328988). For each of the datasets, we generated kmers of 31 bp for each sample as described above. We then searched for the presence of 115 associated kmers from *V. arizonica* using the “filter_kmers” script from the kmer-GWAS pipeline^35^ (https://github.com/voichek/kmersGWAS).

To compare the R-kmers with random sequences in other *Vitis* species, we first extracted 100,000 unique and random kmers from the *V. arizonica* population. We then calculated the frequency of these sequences across all individuals and selected kmers with similar frequencies as the R-kmers mean (0.52), resulting in 38,523 kmers. We then created 100 subsets of 99 random kmers from the set with similar frequencies as R-kmers. Finally, we searched for the presence of each set of other *Vitis* species and calculated the mean frequencies of the 99 kmers in each random set. We report the average and standard error of means across the 100 sets in Fig. 4c.

### Linkage disequilibrium

We calculated the genome-wide LD decay across the *V. arizonica* population with the software PopLDdecay v3.40^94^. We used the filtered SNPs of the 167 individuals from hap1, allowing a maximum distance of 1 Mb (Supplementary Fig. S11). We used the perl script “Plot_OnePop.pl” included in the package to create the decay graph.

To explore the LD landscape of the regions around PdR1 and chromosome 14 as a whole we used Tomahawk v0.7.0 (https://github.com/mklarqvist/tomahawk). We used as input the filtered SNPs of chromosome 14 for hap1 as input, containing the 167 *V. arizonica* accessions. We converted the VCF file into a custom format file (“.twk”) for the package and calculated the LD with the “calc” function. We then filtered LD values using the “view” function, keeping regions with R^2^ > 0.5 and *p* values < 0.001. Given that we were interested in the LD at the chromosome-scale ( ~ 30 Mb) we used the “aggregate” function. Using this function we aggregated R^2^ values in 1000 bins for both the x and y-axis, and used 5 as the minimum cut-off value in the reduction function. Finally, we used the “rtomahawk” R package (https://github.com/mklarqvist/rtomahawk) to create the chomosome-scale LD landscape plot using the aggregated R^2^ values.

### Functional annotation and refinement of *PdR1* gene models

Gene models located within the two haplotypes of *PdR1* were manually refined by visualizing alignments of RNA-seq reads from *V. arizonica* b40-14 leaves^30^ using the Integrative Genomics Viewer (IGV) v.2.4.14^95^. RNA-seq reads were aligned onto the diploid genome of *V. arizonica* b40-14 using HISAT2 v.2.1.0^96^ and the following settings: --end-to-end --sensitive -k 50.

Predicted proteins of *PdR1* genes were scanned with hmmsearch from HMMER v.3.3.1 (http://hmmer.org/) and the Pfam-A Hidden Markov Models (HMM) database^97^ (downloaded on January 29th, 2021). Protein domains with an independent E-value less than 1.0 and an alignment covering at least 50% of the HMM were selected. Transmembrane helices were predicted with TMHMM2 v2.0c^98^. Proteins with a predicted Leucine Rich Repeat (LRR) domain and a transmembrane helix were classified as LRR receptor-like proteins. Proteins having a predicted LRR or lysin motif (LysM), a kinase domain, and transmembrane helices were categorized as receptor-like kinases.

Predicted proteins of *PdR1* genes were aligned onto the predicted proteome of *A. thaliana* and the grape PN40024 (V1 annotation) using BLASTP v.2.2.28 + ^99^. Alignments with an identity greater than 30% and a reciprocal target:query coverage between 75% and 125% were kept. For each *V. arizonica* protein, best hit in the *A. thaliana* and PN40024 proteomes was determined using the highest product of identity, query coverage, and reference coverage. The sequences of the two ORFs (V.ari-RGA14 and V.ari-RGA18)^22^ were aligned onto the b40-14 genome using blastn v. 2.2.28 + ^99^.

### Gene expression analyses

To evaluating the transcript abundance of candidate gens, plants from three PD-resistant genotypes and three PD-susceptible genotypes of the 07744 population ([*V. rupestris* Wichita refuge x *V. arizonica* b40-14] x *V. vinifera* Airen) were propagated in a controlled environment and inoculated with either *Xylella fastidiosa* or water. Pieces of green stem at 10, 20, 30 and 40 cm above the inoculation were collected from each plant at 1, 2, 3, and 4 weeks postinoculation. Pieces of the green stem from each genotype were pooled together. Each genotype constitutes a biological replicate. All plant material was immediately frozen in liquid nitrogen after collection and ground into powder. Total RNA were extracted as described^100^, using the Spectrum Plant Total RNA kit (Sigma-Aldrich). RNA quality and quantity were determined using a Nanodrop 2000 spectro- photometer (Thermo Fisher Scientific) and a Bioanalyzer Chip RNA 7500 series II (Agilent Technologies). cDNA libraries were prepared using the Illumina TruSeq RNA sample preparation kit v.2 (Illumina, CA, USA) and sequenced in single-end 100-bp reads on an Illumina HiSeq4000. RNA-seq reads were parsed using Trimmomatic v.0.3^73^ with the following settings: LEADING:7 TRAILING:7 SLIDINGWINDOW:10:20 MINLEN:36. Transcript abundance was evaluated with Salmon v.1.5.1^101^ with the parameters: --gcBias --seqBias --validateMappings. A transcriptome index file was built using a k-mer size of 31 and the combined transcriptomes of *V. arizonica* b40-14 (v2.1), *V. vinifera* cv. Cabernet Sauvignon^70^, with their genomes as decoy. Quantification files were imported using the R package tximport v.1.20.0^102^. The RNAseq data were deposited in NCBI Bioproject PRJNA956994.

### Bioclimatic variables associated with resistance

To identify the association between SNPs associated with resistance and the environmental landscape, we applied gradient forest (GF)^103^, which models the turnover in genetic composition across the landscape^45^ and identifies both the bioclimatic variables that contribute to the construction of the model and the ‘turnover function’ - i.e., the change of genetic composition across the landscape^45,104,105^. To estimate the GF model, we used the *gradient forest* package in R, using the 25 SNPs identified by LFMM2 and EMMAX as response variables and using bioclimatic variables as predictive variables. The 19 bioclimatic variables were filtered to retain any correlations < 0.80, based on a variance inflation factor calculated by corSelect from the R package *fuzzySim*^106^. After filtering, we retained 10 of 19 bioclimatic variables (BIO1, BIO2, BIO3, BIO4, BIO8, BIO9, BIO12, BIO14, BIO17, BIO19). We performed the GF analysis using SNP frequencies from each individual (i.e., 0, 1 and 0.5 for heterozygotes)^59^ and repeated the analysis 1000 times. We also plotted the turnover functions for each bioclimatic variable to show how populations with different resistance to PD are distributed across allele frequency change (Supplementary Fig. S15).

### Predicting PD resistance

To determine whether genetic and environmental variables predicted PD resistance, we first ran a linear model individually for each bioclimatic and genetic variable to determine their individual predictive power, using the *lm* function in R. We performed 1000 bootstrap replicates for each model to estimate the variance in predictive ability. For the bioclimatic variables, we estimated the individual linear models between the 10 bioclimatic values from where the *arizonica* individuals were sampled and their bacterial load. For some genetic variables, we first estimated a polygenic score across SNPs that associated with bacterial load. This polygenic score, which we called the PD score (*S*_*pd*_), was calculated as the proportion of alleles that contribute to resistance to PD in a given individual. The state of these contributing alleles was inferred from the GWA results. *S*_*pd*_ was designed after the population adaptive index, which measures the proportion of alleles that show patterns of local adaptation^107^. *S*_*pd*_ assumes that alleles contribute equally to PD resistance and that a higher proportion of resistance alleles correlates with lower CFU in the individuals*. S*_*pd*_ ranges from 0 when no resistance alleles are present to 1 when all alleles across loci are homozygous for the resistant state. We estimated *S*_*pd*_ for different sets of SNPs: *i*) all candidate SNPs across the genome (25 SNPs), *ii*) all candidate SNPs on chromosome 14 (16 of the 25 SNPs); *iii*) all candidate SNPs in the region defined by *PdR1* (10 of 25 SNPs); *iv*) all candidate SNPs on chromosome 15 (6 of 25 SNPs). To control for potential ancestry effects, we also tested an analogous *S*_*pd*_ score, which we called the reference PD scores (*R*_*pd*_). For *R*_*pd*_ we sampled 25 random reference SNP (SNPs that were not significant for any of the two GWA methods) and obtained a *R*_*pd*_ distribution based on 1000 *R*_*pd*_ values. Following the concept of *S*_*pd*_, we also estimated a Kmer score (*K*_*pd*_), which consisted of the proportion of Kmers associated with resistance across populations. A value of 1 indicates that the individual has all the resistant Kmers and none of the susceptible Kmers (see Above), while a value of 0 indicates that the individual has all the susceptible kmers and none of the resistant ones. Finally, we also estimated a CNV score (*C*_*pd*_) that corresponds to the number of adaptive copy variants in an individual, where a higher number of CN variants indicates that the individual is more resistant to PD. For the *S*_*pd*_, *K*_*pd*_ and *C*_*pd*_ scores, we estimated the fit between the scores and the bacterial load. Finally, for the genetic independent variables, we also analyzed the linear model between the assignments into genetic groups (K1 and K2) based on the admixture analyses and the concentration of bacterial load.

### Climate modeling

We used BIO8 to model the future distribution of *Xylella fastidiosa*, assuming that 8 °C and 10 °C were predictive thresholds of potential *Xylella fastidiosa* presence and absence. We predicted two discrete types of locations: i) regions across the globe that currently have BIO8 > 8 °C or >10 °C in the present but predicted to have BIO8 < 8 °C and <10 °C in the future and ii) regions across the globe that currently have BIO8 < 8 °C and <10 °C in the present but predicted to have BIO8 > 8 °C and >10 °C in the future. To make these predictions, we downloaded the BIO8 data at a 2.5 minutes resolution from Worldclim 2^108^ for the present and for 54 climatic models in the future to consider the uncertainty in future climate projections. These future climate models included five global circulation models (GFDL-ESM4, IPSL-CM6A-LR, MPI-ESM1-2-HR, MRI-ESM2-0, UKESM1-0-LL), three-time periods (2041-2060; 2061-2080; 2081-2100) and 4 shared socioeconomic pathways (SSPs: 126, 245, 370, 585). To plot the areas where the climate is expected to change across the 10 °C threshold, for the present and the 54 future layers we set the raster layers to 1 and 0 if they were above or below the BIO8 temperature threshold. Next, we subtracted each future layer from the present layer. Areas with values of -1 indicate that a region in the future layer is expected to be under the threshold but is currently above the threshold. Regions with values of 0 indicated that the threshold remained unchanged in present and future layers. Finally, regions with values of +1 indicated that the future layer was expected to become above the threshold in the future. We performed this calculation for the 54 layers (or models) and created a sum_raster object by summing the 54 raster layers.

We tested how BIO8 will change in the future for regions where *V. arizonica, V. vinifera* (grapevines), *Citrus* sp., *Olea europea* (olives), *Prunnus amygdalus* (almond) and *Coffea* sp currently grow. For this, we first used the gbif function in the R dismo package^109^ to download all the known locations of V *arizonica*, grapevines, olives, almonds, coffee sp. and *Citrus* sp (gbif.org; download data: 2022-06-06). Next, for each species we used the CoordinateCleaner package in R^110^ to remove locations that 1) were duplicated; 2) had equal longitude and latitude; 3) were next to country centroids, capitals of countries, biological stations or gbif headquarters; 4) were in the sea; 5) were outliers based on the “quantile” option. Finally, we also removed locations if they were the only report in a given country, suggesting they may be outliers. After cleaning the data, we retained 6204 locations (from 11,834) for *Coffee* sps.; 3386 locations (from 9992) for almonds; 1111 locations (from 1155) for *V. arizonica*; 5256 locations (from 7853) for *Citrus* sps.; 174,713 locations (from 204,775) for olives; and 33,225 locations (from 47,075) for grapevines. For each species, we used the extract function in the raster R package^111^ to obtain for each location whether climate is expected to cross the 8 °C and 10 °C BIO8 threshold. For each species we estimated the percentage of locations that are expected to move below and above the 8 °C and 10 °C threshold across the 54 layers.

## Supplementary information

Supplmentary Information

Description of Additional Supplementary Files

Supplementary Data 1-10

## Data Availability

Raw genome resequencing data of the 147 new *V. arizonica* accessions and the PD resistance *V. vinifera* breeding lines are available at NCBI under BioProfect ID PRJNA842753. The remaining 20 V. arizonica accessions, which were previously published, are available at NCBI under BioProject PRJNA731597. The RNAseq data were deposited in NCBI Bioproject PRJNA956994.The published long-read *V. arizonica* genome sequencing data were taken from NCBI BioProject ID PRJNA593045. The genome files and a genome browser can also be found at www.grapegenomics.com/pages/Vari/.

## Code availability

Analyses in this paper relied on published methods, the sources for which has been cited throughout. Climate change analyses relied on previously published pipelines^46^, which can be accessed here: https://static-content.springer.com/esm/art%3A10.1038%2Fs41559-021-01526-9/MediaObjects/41559_2021_1526_MOESM1_ESM.pdf.
