# Supplementary material for: Multigenic resistance to Xylella fastidiosa in wild grapes (Vitis sps.) and its implications within a changing climate
Source: Commun Biol. 2023 May 30;6:580. doi: 10.1038/s42003-023-04938-4 (PMC10229667; doi:10.1038/s42003-023-04938-4)
Supplement: Supplementary file 1 — Supplmentary Information [file 42003_2023_4938_MOESM1_ESM.pdf]

Multigenic resistance to *Xylella fastidiosa* in wild grapes (*Vitis* sps.) and its implications within a changing climate

Abraham Morales-Cruz, Jonas Aguirre-Liguori, Mélanie Massonnet, Andrea Minio, Mirella Zaccheo, Noe Cochetel, Andrew Walker, Summaira Riaz, Yongfeng Zhou, Dario Cantu, Brandon Gaut.

Correspondence to: bgaut@uci.edu

This PDF includes:

Figs. S1 to S20

SUPPLEMENTAL FIGURES

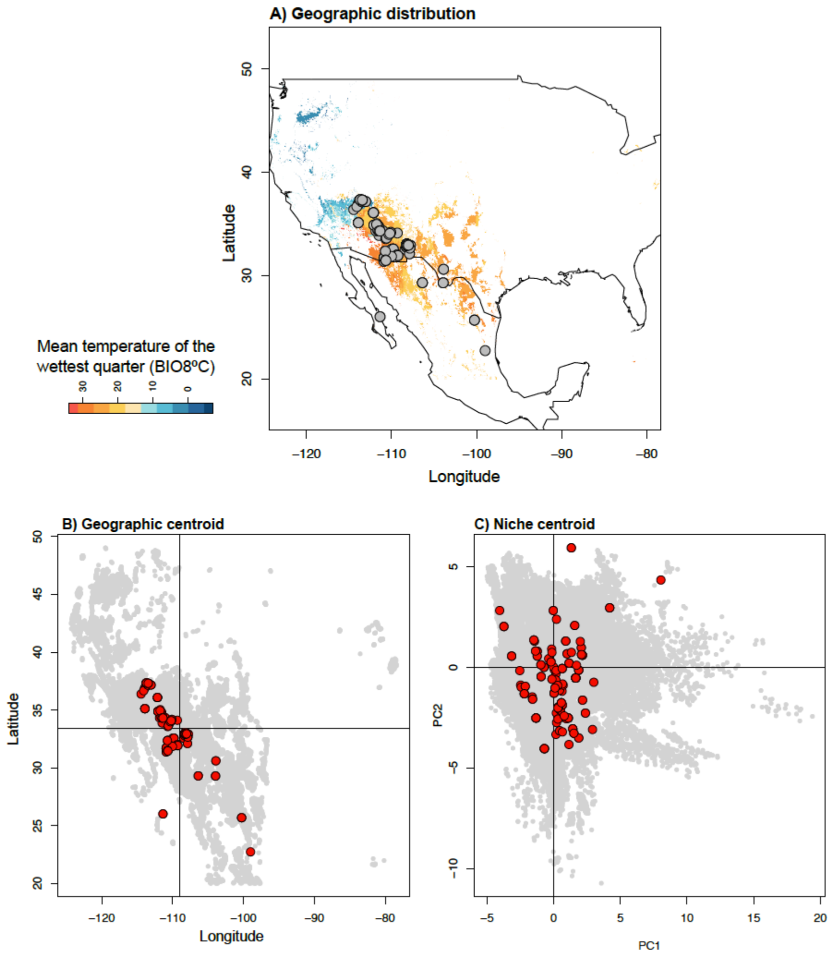

Fig. S1.

Geographic locations of *V. arizonica* samples used in this study (dots in each graph) illustrate a reasonable geographic distribution compared to A) the Species Distribution Model (SDM) projections for extant environments where the species could exist in theory based on BIO8, B) the predicted geographic centroid of the species and C) the predicted niche centroid of the species. The generation of these maps relied on information from WorldClim2 (https://www.worldclim.org/) and CMIP6 (https://pcmdi.llnl.gov/CMIP6/).

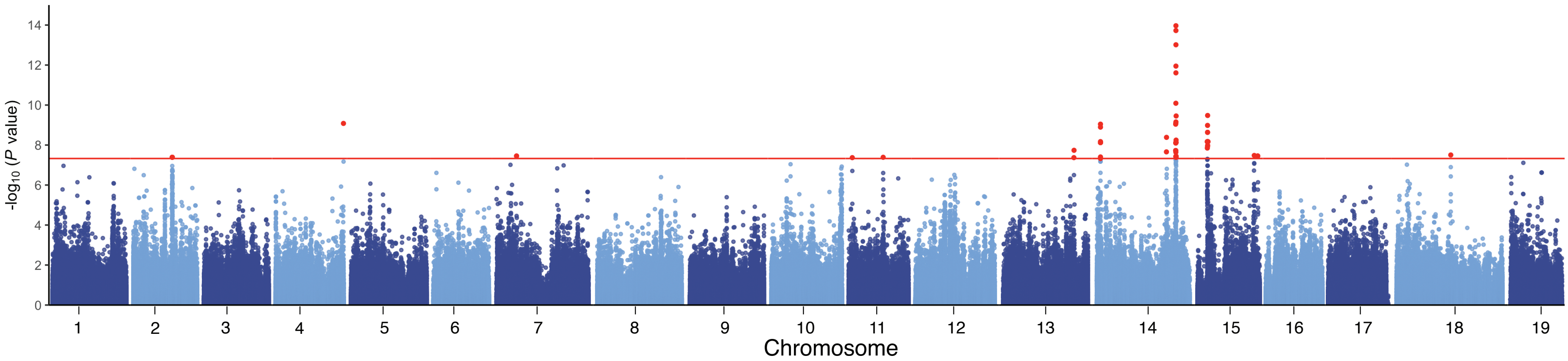

Fig. S2.

Manhattan plot from the genome-wide association studies of SNPs predicted in hap1 with *X. fastidiosa* loads (CFU/ml) performed by LFMM2. The red line indicates Bonferroni adjusted p-value of 0.05, which was used as the significance threshold.

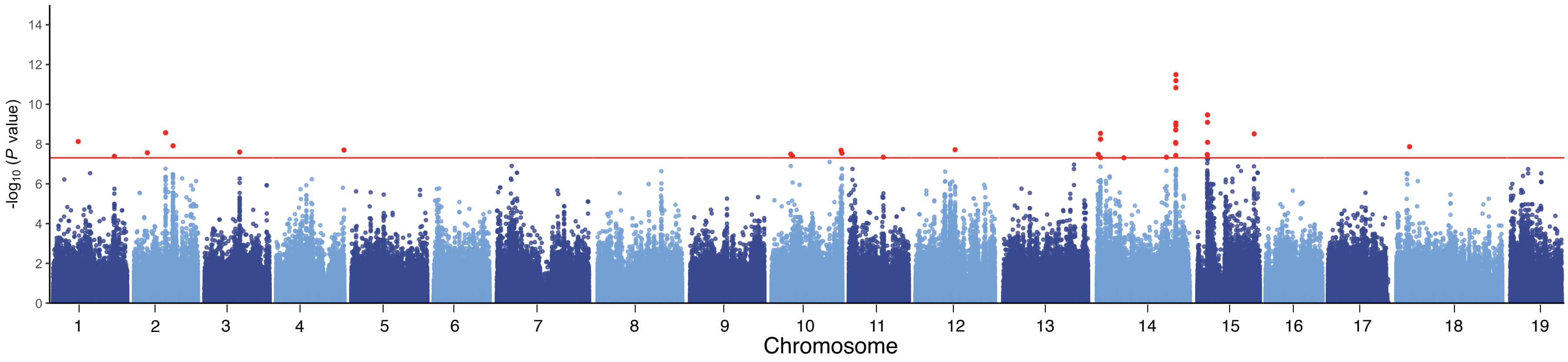

Fig. S3.

Manhattan plot from the genome-wide association studies of SNPs predicted in hap1 with *X. fastidiosa* loads (CFU/ml) performed by EMMAX. The red line indicates Bonferroni adjusted p-value of 0.05, which was used as the significance threshold.

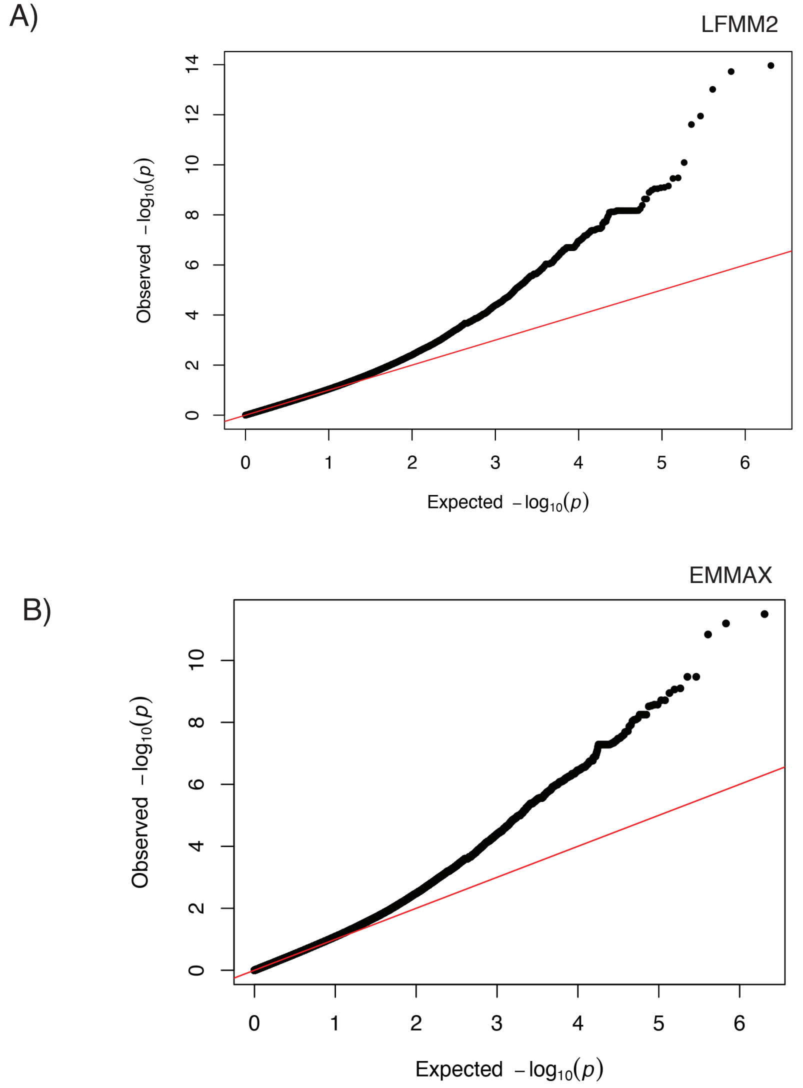

Fig. S4.

Q-Q plot of p-values for the GWAS performed by A) LFMM2 and B) EMMAX on hap1.

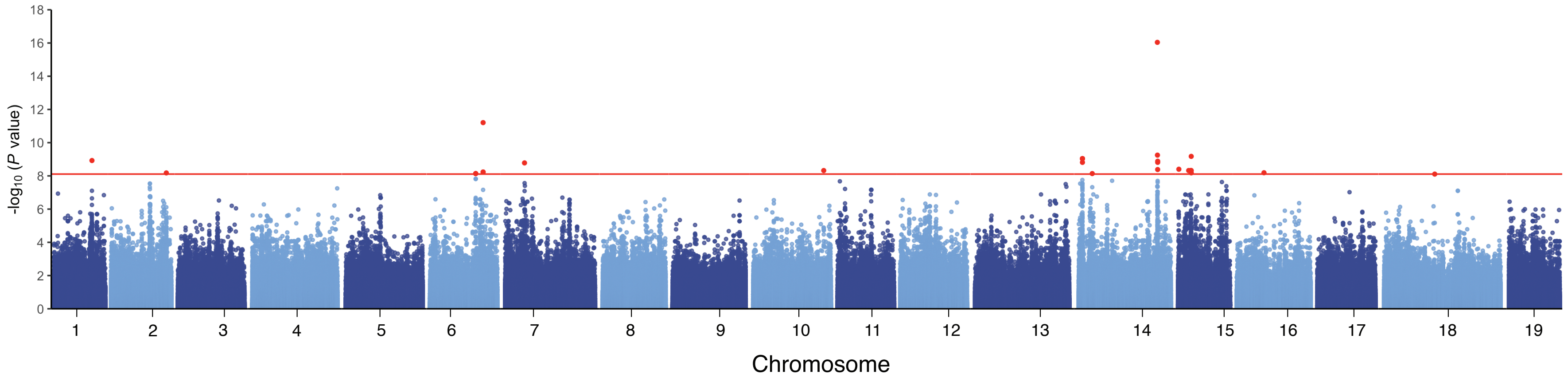

Fig. S5.

Manhattan plot from the genome-wide association studies of SNPs predicted in hap2 and *X. fastidiosa* loads (CFU/ml), as performed by LFMM2. The red line indicates Bonferroni adjusted p-value of 0.05, which was used as the significance threshold.

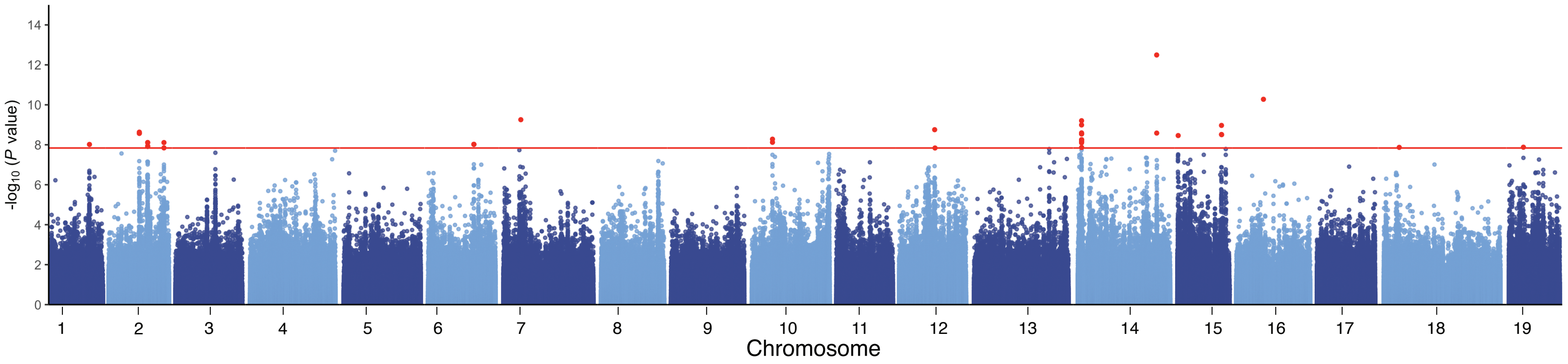

Fig. S6.

Manhattan plot from the genome-wide association studies of SNPs predicted in hap1 and *X. fastidiosa* loads (CFU/ml) performed by EMMAX. The red line indicates Bonferroni adjusted p-value of 0.05, which was used as the significance threshold.

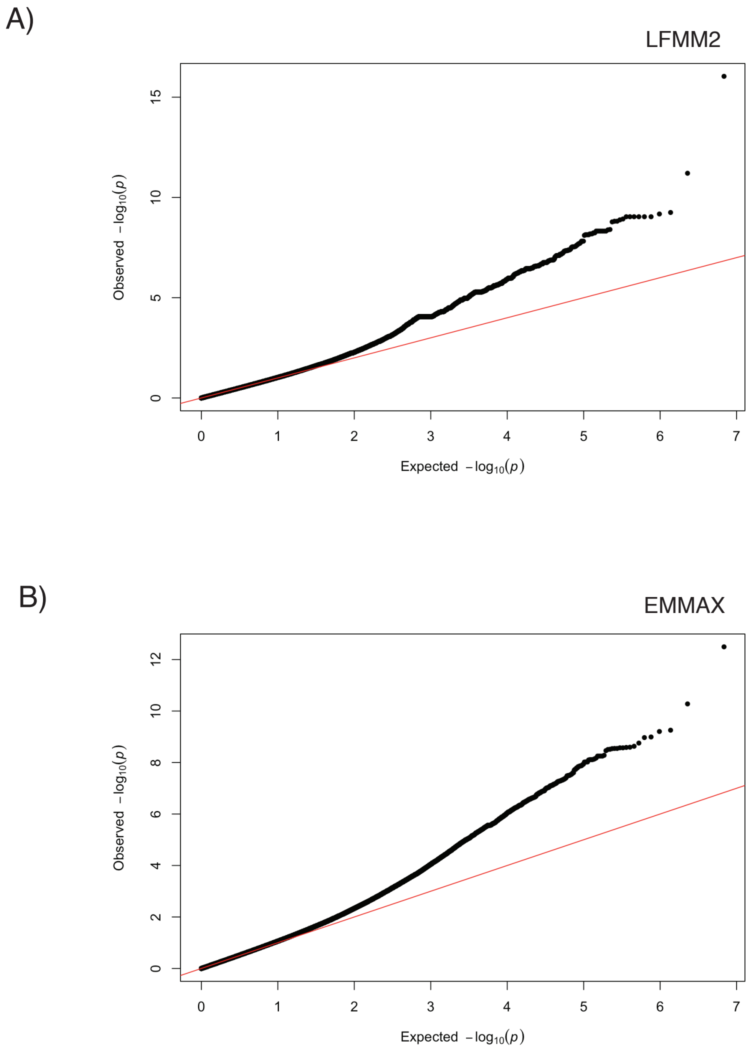

Fig. S7.
Q-Q plot of p-values for the GWAS in hap2 performed by A) LFMM2 and B) EMMAX.

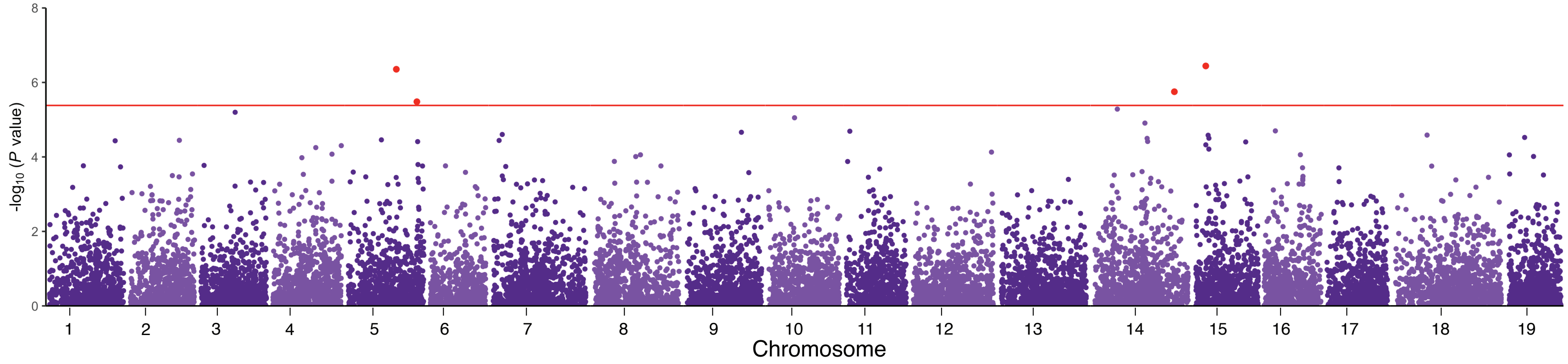

Fig. S8.

Manhattan plot from the genome-wide association studies of Copy Number Variants (CNVs) predicted in hap1 and *X. fastidiosa* loads (CFU/ml). The red line indicates Bonferroni adjusted p-value of 0.05, which was used as the significance threshold.

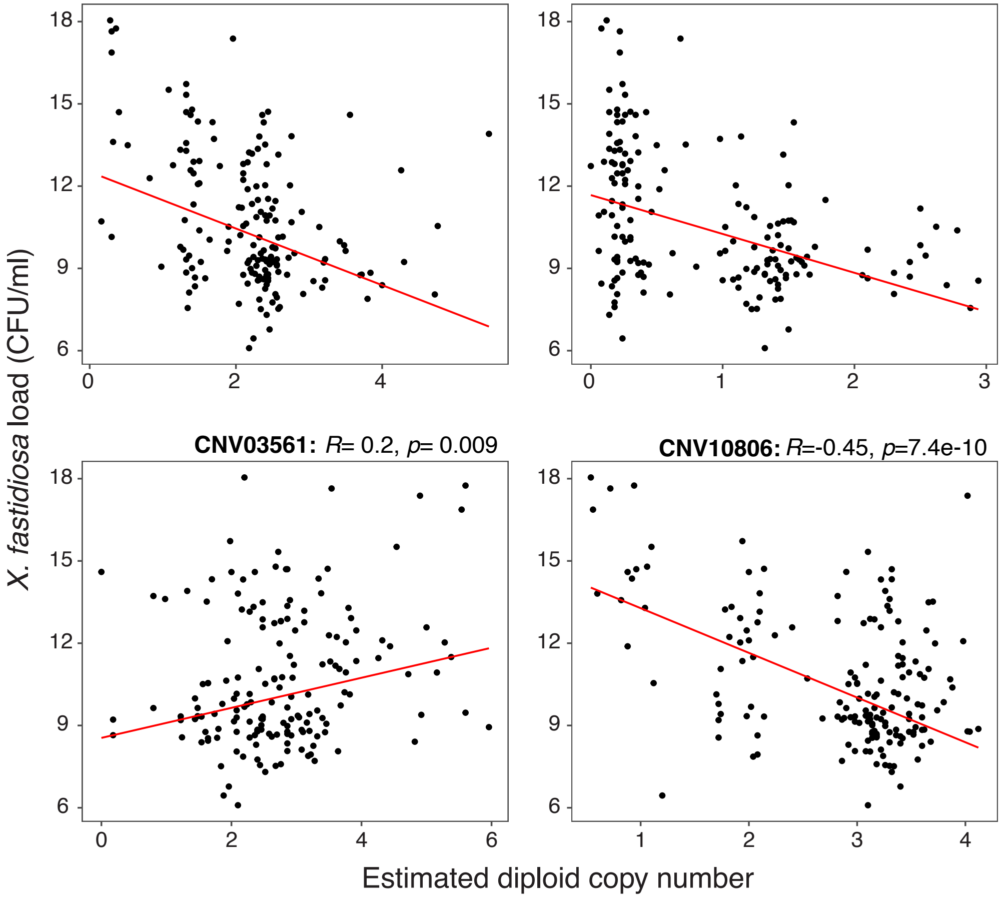

Fig. S9.

Scatterplots of four CNVs significantly associated with *X. fastidiosa* loads. Each dot represents an individual, with its estimated load (y-axis) and estimated diploid copy number (x-axis). Negative slopes indicate that higher copy numbers are associated with higher resistance.

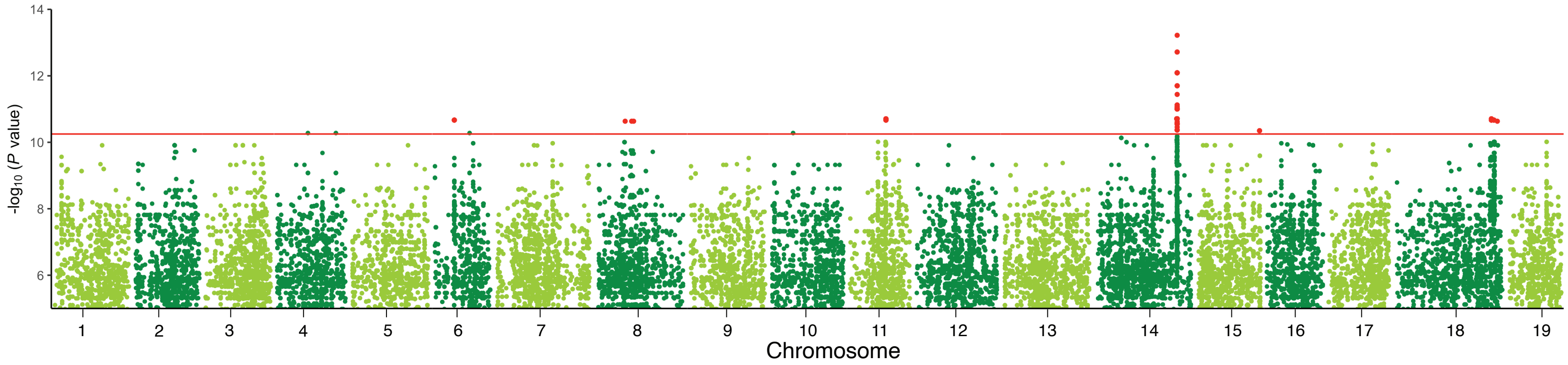

Fig. S10.

Manhattan plot from the genome-wide association studies of kmers with *X. fastidiosa* loads (CFU/ml). The plot shows kmers mapping to hap1 with a maximum of one mismatch. The red line indicates Bonferonni adjusted p-value of 0.05, which was used as the significance threshold.

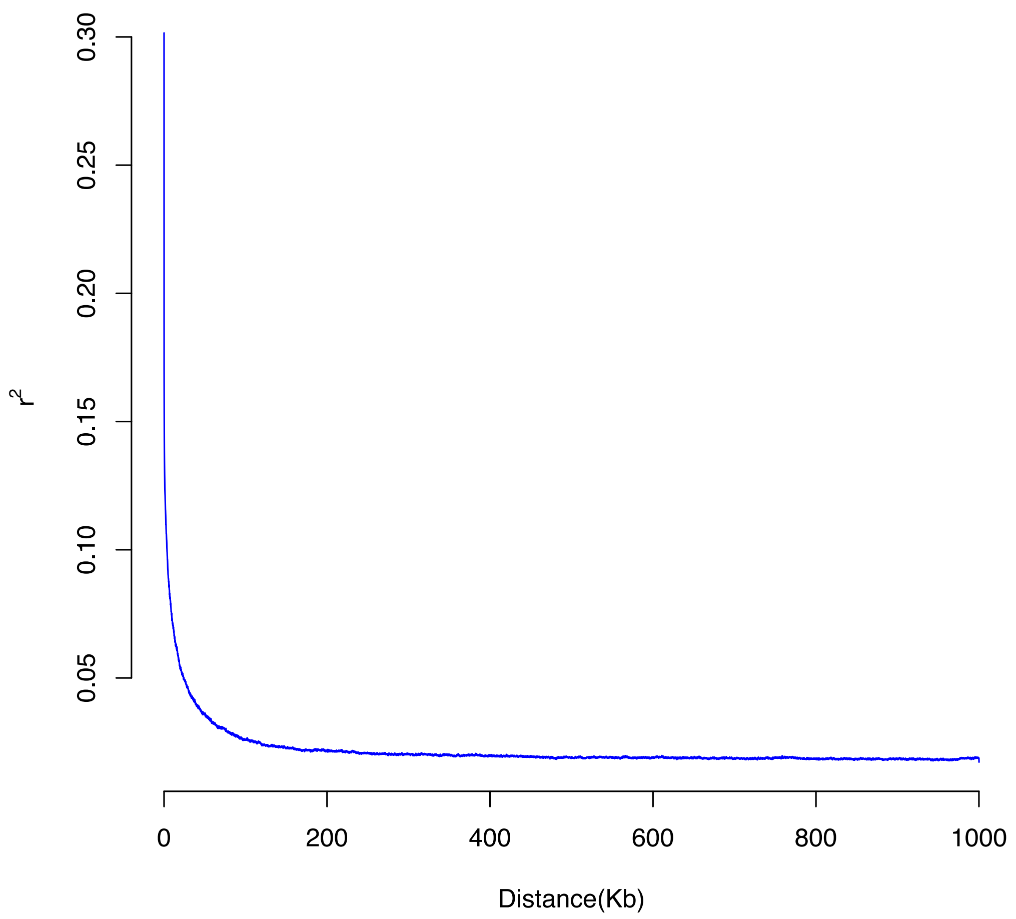

Fig. S11.
Estimated **l**inkage disequilibrium decay of genome-wide SNPs from the 167 *V. arizonica* accessions.

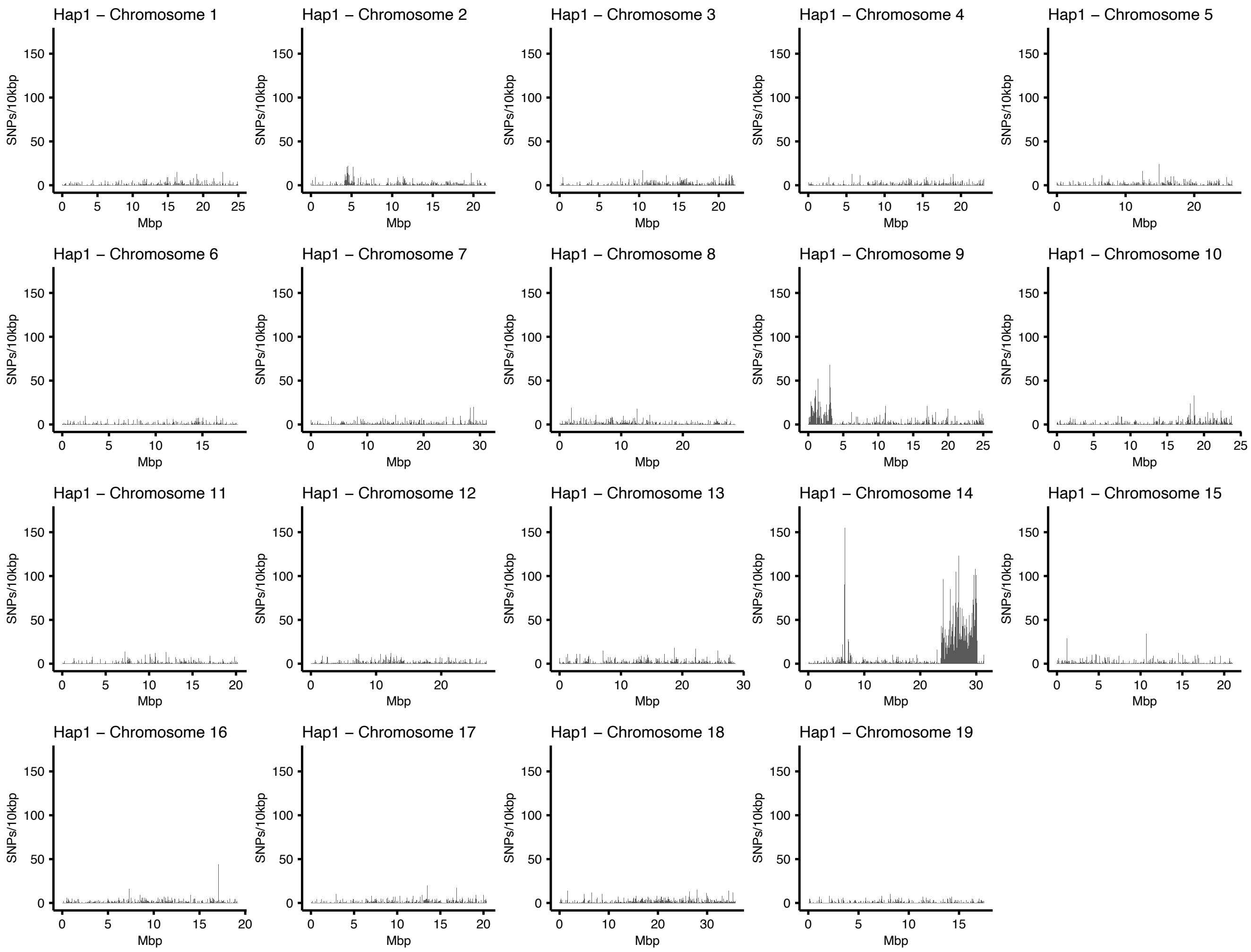

Fig. S12.

Plots of the 19 chromosomes, based on heterozygosity across 5 varietals backcrossed for PD resistance. The peaks represent the number of heterozygous SNPs across 10kb windows in which one of the heterozygous alleles was contributed by *V. arizonica*.

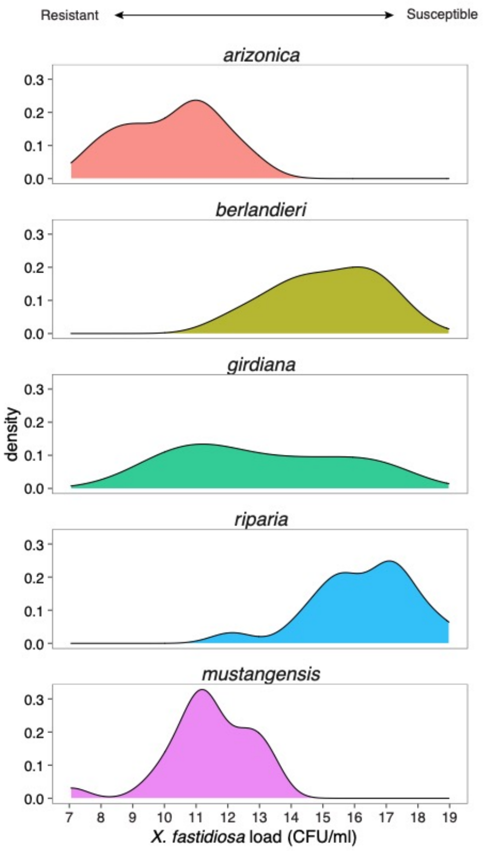

Fig. S13.
Distribution of *X. fastidiosa* loads (CFU/ml) in evaluated individuals of six wild grape species. Sample sizes for each species are provided in Fig. S14.

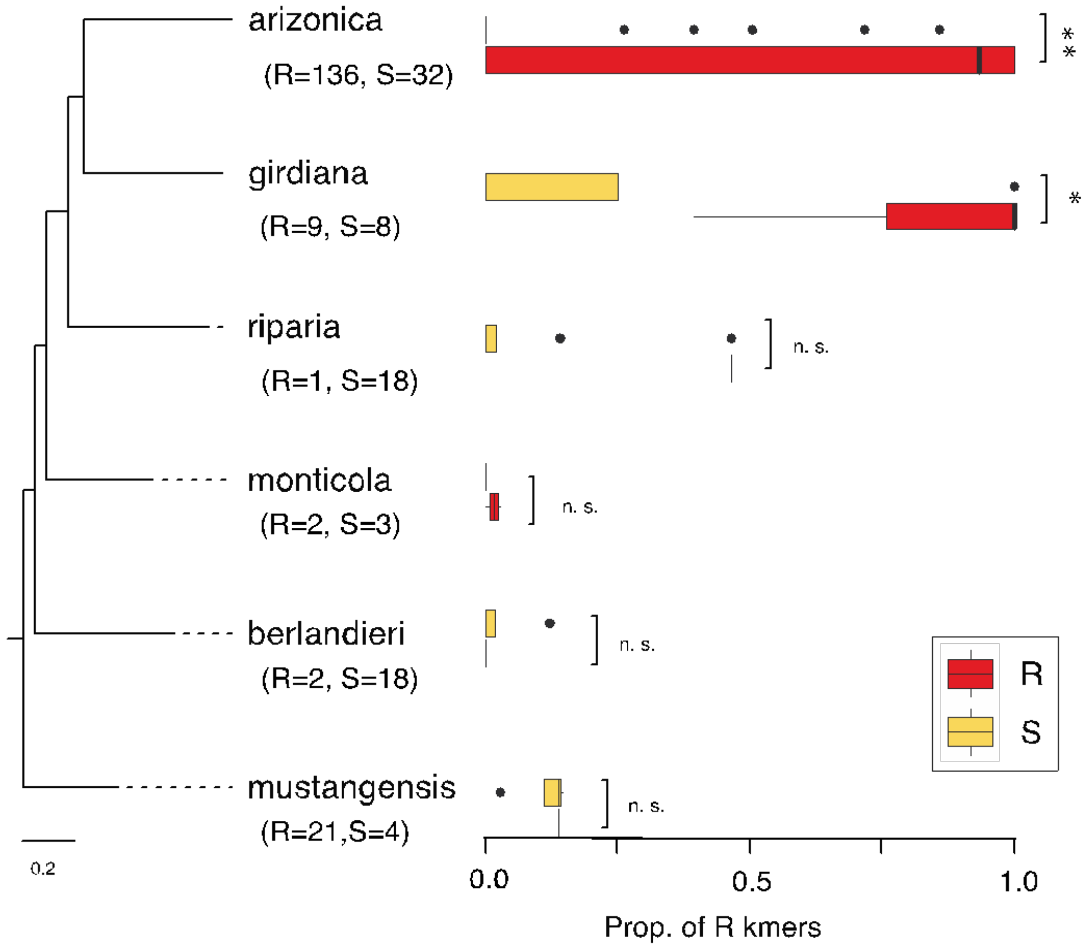

Fig. S14.

The frequencies of R-kmers in resistant (in red) and susceptible (yellow) individuals across six wild grape species. The sample sizes for resistant (R) and susceptible (S) individuals in each sample is provided in the paranthesis under species name. The inferred phylogeny of the species is provided to the left. Both *V. arizonica* and *V. girdiana* have significant differences in the frequency of R-kmers between R and S individuals, as indicated by asterisks on the right, but the other species do not have significant differences for R-kmers between R and S individuals. In fact, R-kmers are found less often than expected in *V. riparia*, *V. monticola*, *V. berlandieri* and *V. mustangensis* than control kmers chosen to have similar population frequencies in *V. arizonica* as R-kmers (Fig. 4C).

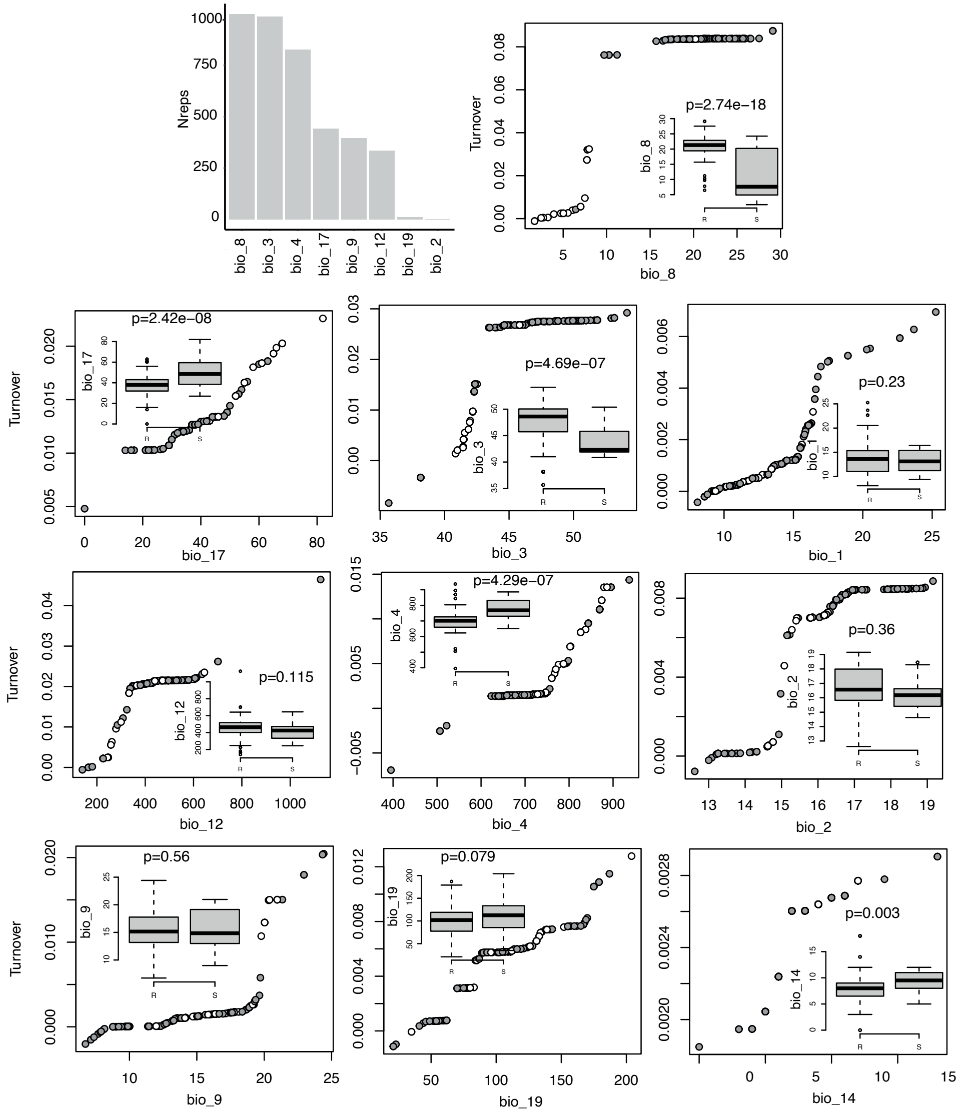

Fig. S15.

Results from Gradient Forest analyses. The top left graph is a histogram of the number of times, out of 1000 GF runs, that a particular bioclimatic variable was found to be among the top-3 most important variables. The remaining graphs show the turnover function for each of the 10 bioclimatic variables included in GF analyses, based on a single GF run. For each turnover graph, the x-axis is the distribution of the variable across sample sites (e.g., in degrees C for bio3 and several other variables.)

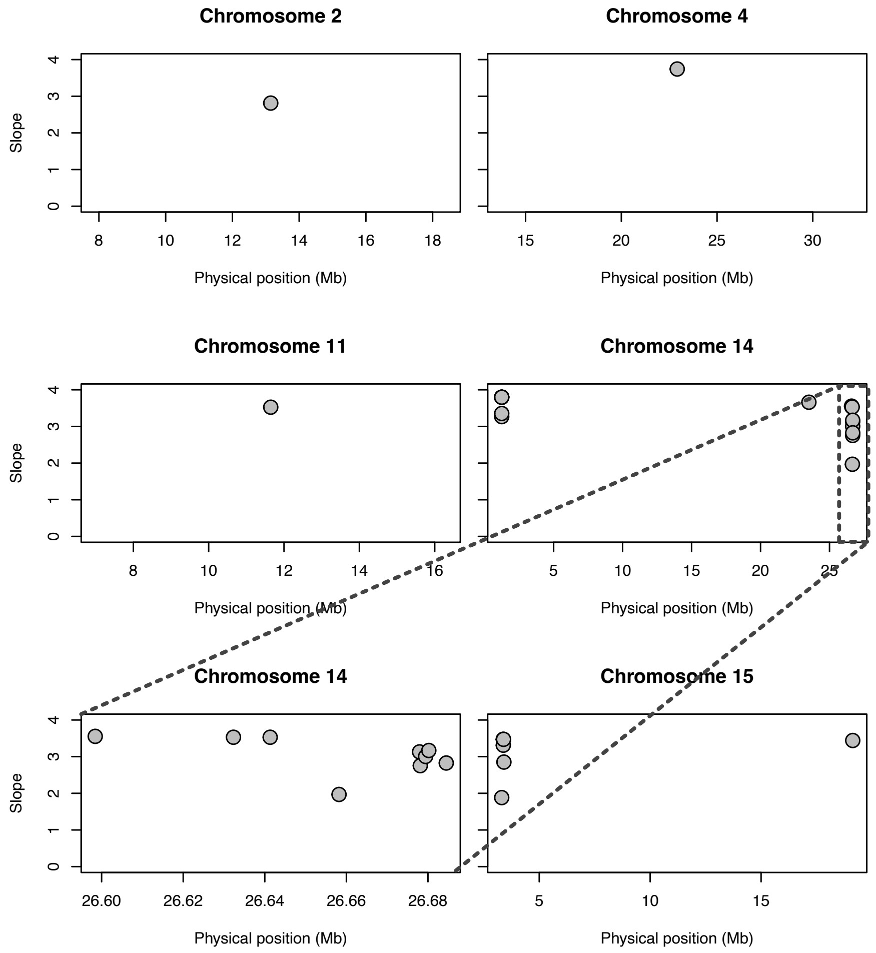

Fig. S16.
Effect sizes for each of the 25 outlier SNPs that showed significant associations with PD resistance based on LFMM2 and EMMAX. The plot shows in the x-axis the physical position of each SNP in a given chromosome. The y-axis shows the absolute slope value of the linear model between the genotypes (independent variable) and the phenotypes (response variable). We also show a zoom in chromosome 14, indicating the area where Pdr1 is found.

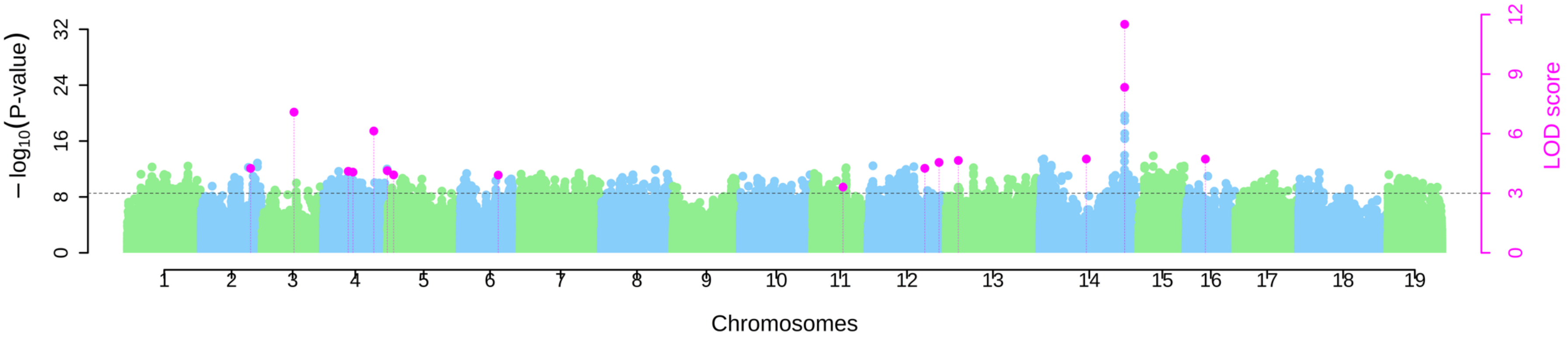

Fig. S17.

Manhattan plot from the multi-locus mixed-model genome-wide association studies of SNPs predicted in hap1 and *X. fastidiosa* loads (CFU/ml) performed by mrMLM.

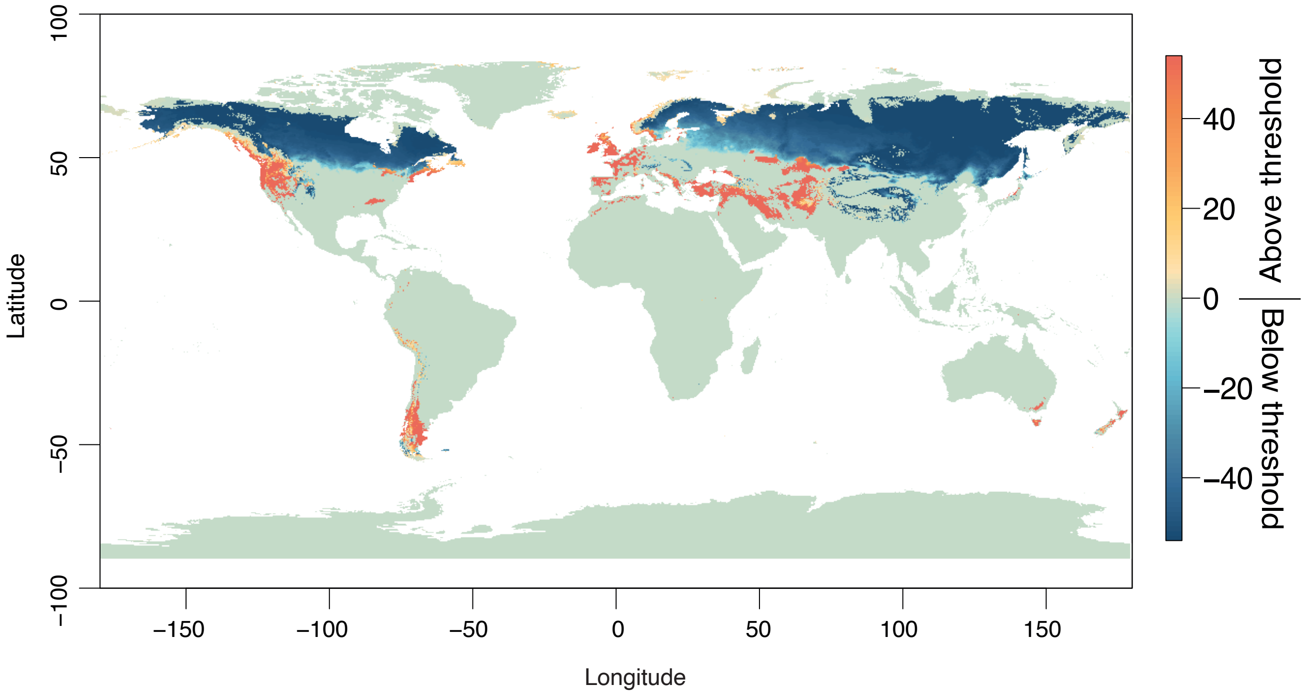

Fig. S18.
Climate predictions and projections of the prevalence of Xylella fastidiosa for focal crops. The map portrays the number of climate models (out of 54 total) that support movement across the BIO8 = 8ºC threshold. The warmer colors reflect regions that are moving from below (in the present) to above the threshold, while the cooler colors portray areas that are moving from above (in the present) to below the threshold. The generation of these maps relied on information from WorldClim2 (https://www.worldclim.org/) and CMIP6 (https://pcmdi.llnl.gov/CMIP6/).

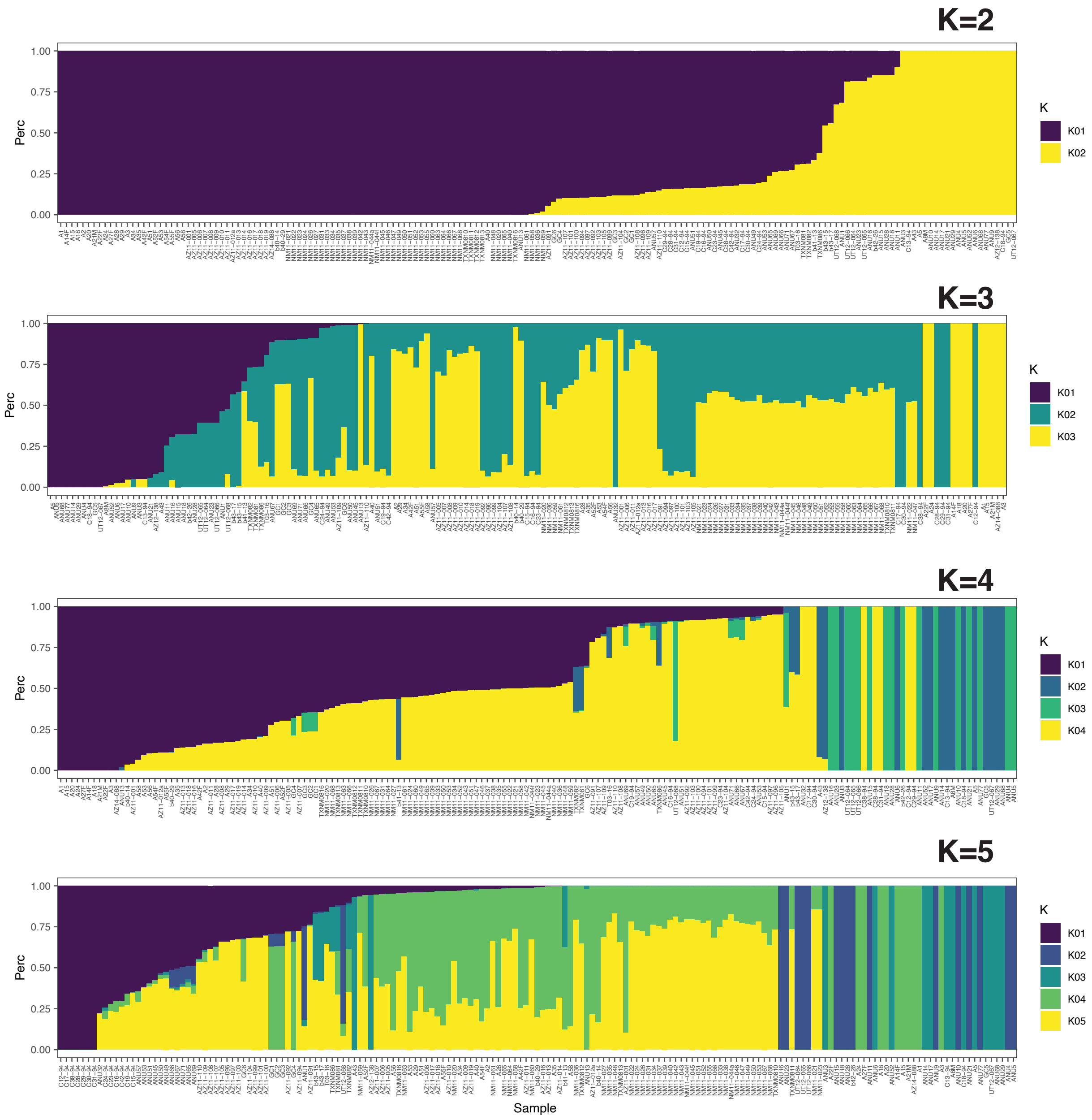

Fig. S19.

Genetic structure of *V. arizonica* with grouping values (*K*) of 2 to 5. The best grouping corresponds to K=2.

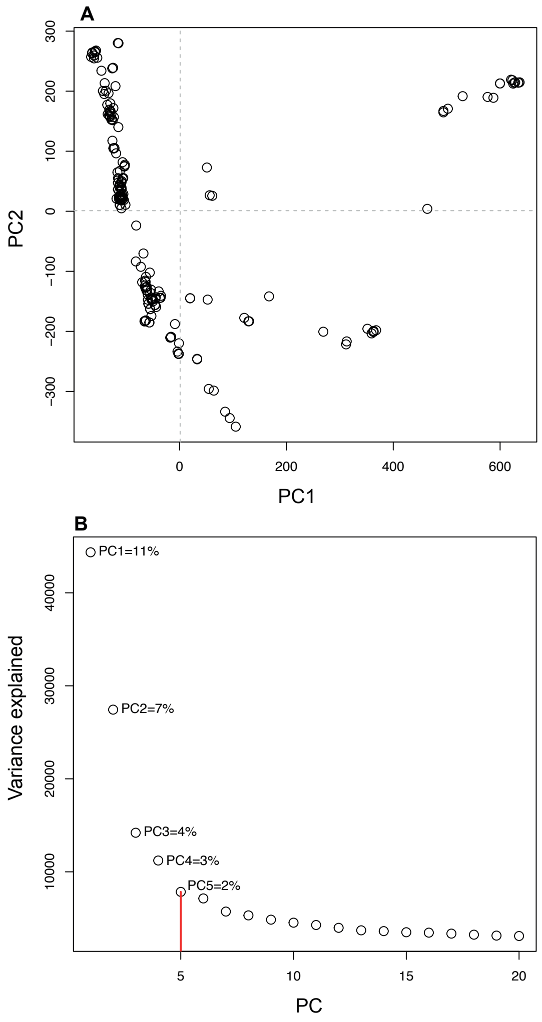

Fig. S20.
individual structure characterized using Principal component analysis. A) First two principal components showing the scores of each individual sampled. B) Screeplot showing the variance explained by the first 20 PCs. The knee in the plot at PC=5 suggest that 5 latent factors (red line) should be used in the LFFM2 analysis. The plot also shows the percentage of variance explained by the first 5 PCs.
