# Supplementary material for: Multigenic resistance to Xylella fastidiosa in wild grapes (Vitis sps.) and its implications within a changing climate
Source: Commun Biol. 2023 May 30;6:580. doi: 10.1038/s42003-023-04938-4 (PMC10229667; doi:10.1038/s42003-023-04938-4)
Supplement: Supplementary file 2 — Description of Additional Supplementary Files [file 42003_2023_4938_MOESM2_ESM.pdf]

Description of Additional Supplementary Files

File name: Supplementary Data S1
Description: Quantitative concentrations in CFU/ml of X. fastidiosa detected in the V. arizonica accessions used in this study.

File name: Supplementary Data S2
Description: Significant SNPs from the GWAS in hap1 and hap2 and from both methods.

File name: Supplementary Data S3
Description: CNVs detected around the SNP-defined peaks associated with PD resistance.

File name: Supplementary Data S4
Description: Name, sequences, p-values and adjusted p-values of the 115 significant kmers.

File name: Supplementary Data S5
Description: Best hit mapping information of significant kmers to hap1, hap2 and unplaced contigs.

File name: Supplementary Data S6
Description: Gene functional annotation of genes around PD-associated peaks.

File name: Supplementary Data S7
Description: Presence-absence matrix of significant kmers in V. arizonica.

File name: Supplementary Data S8
Description: Presence-absence matrix of significant kmers in other species.

File name: Supplementary Data S9
Description: Gene expression of the homologous genes of V.ari-RGA14 and V.ari-RGA18 (Agüero et al., 2022) in V. arizonica b40-14. Transcript abundance is shown as Transcripts per Million (TPM). n = three genotypes for stems. n = 1 for b40-14 leaves.

File name: Supplementary Data S10
Description: Significant SNPs from the multi-locus mixed-model GWAS on hap1.
